# Acute Endoplasmic Reticulum Stress Induces Inflammation Reaction, Complement System Activation, and Lipid Metabolism Disorder of Piglet Livers: A Proteomic Approach

**DOI:** 10.3389/fphys.2022.857853

**Published:** 2022-04-13

**Authors:** Xiaohong Wang, Hairui Xin, Mingjie Xing, Xianhong Gu, Yue Hao

**Affiliations:** State Key Laboratory of Animal Nutrition, Institute of Animal Sciences, Chinese Academy of Agricultural Sciences, Beijing, China

**Keywords:** endoplasmic reticulum stress, lipid metabolism, complement system, inflammation, pig liver, tunicamycin, liver injure

## Abstract

Endoplasmic reticulum stress (ERS) is closely associated with the occurrence and development of many liver diseases. ERS models mostly include experimental animals such as rats and mice. However, pigs are more similar to humans with regards to digestion and metabolism, especially liver construction, yet few reports on ERS in pigs exist. In order to explore changes in the liver under ERS, we used tunicamycin (TM), which can cause liver jaundice and damage liver function, to establish acute ERS models in piglets using a low TM dosage (LD, 0.1 mg/kg body weight (bw)), high TM dosage (HD, 0.3 mg/kg bw), or vehicle for 48 h. We found that both LD- and HD-induced ERS, as verified by the ERS-linked proteins. Furthermore, the concentrations of the proinflammatory cytokines, namely, TNF-α and IL-6 were elevated in TM-treated piglet livers, and the plasma levels of IL-6 and CRP were also higher, indicating the occurrence of inflammation in TM-treated piglets. The complement system was activated in TM-treated piglets, as indicated by increased levels of complement factors and activation products C3, C5a, and AP50. In order to gain insights into the global changes in liver proteins under ERS, we performed an isobaric tag for relative and absolute quantitation (iTRAQ)-based proteomic analysis on the livers of HD- and vehicle-treated piglets. Proteomic analysis identified 311 differentially expressed proteins (DEPs) between the two groups, and a Kyoto Encyclopedia of Genes and Genomes (KEGG) pathway analysis suggested that the DEPs were mainly enriched in signaling pathways such as metabolic pathways, protein processing in the endoplasmic reticulum, and complement and coagulation cascades. Many proteins involved in protein folding, lipid transport, and oxidation were upregulated. Proteins involved in lipid synthesis were downregulated to alleviate liver steatosis, and most complement factors were upregulated to protect the body, and Pearson correlation analysis found that most of the DEPs in the complement and coagulation pathway were significantly correlated with plasma CRP, IL6 and AP50. Our results revealed that TM can activate ERS, marked by liver injury and steatosis, inflammatory reactions, and complement activation in piglets.

## 1 Introduction

Endoplasmic reticulum (ER) stress (ERS) is a physiological and pathological process in which ER function is disturbed for various reasons. Liver cells contain large amounts of ER, which is involved in regulating protein homeostasis. During ERS activation, protein synthesis in hepatocytes is blocked, and intracellular homeostasis is disrupted, causing hepatic pathologies. ERS is linked to liver damage caused by various clinical liver diseases, including non-alcoholic fatty liver disease (NAFLD), non-alcoholic steatohepatitis (NASH), and cirrhosis. Under ERS, the unfolded protein response (UPR) is activated by three classic pathways including inositol-requiring enzyme 1 (IRE1), activating transcription factor 6 (ATF6), and protein kinase R-like endoplasmic reticulum kinase (PERK) to reduce the endoplasmic reticulum load and the amount of unfolded and misfolded proteins to maintain homeostasis (Gardner et al., 2013).

The significance of the UPR in the pathogenesis of NAFLD has been confirmed by gene knockout studies. In one study, the three sensory pathways were deleted, and the hepatic response to ERS was affected, leading to hepatic steatosis (Rutkowski et al., 2008). In patients with metabolic syndrome, NASH has been found to be associated with IRE1α deficiency (Puri et al., 2008). However, persistent or excessive ERS damages the structure and function of the ER, activates the inflammatory response, and triggers a series of pathological reactions leading to tissue damage, although inhibition of the inflammatory response can ameliorate the tissue damage induced by ERS (Li et al., 2021). Studies have shown that complement activation is accompanied by an inflammatory response and tissue damage (McCullough et al., 2018). Chronic inflammation contributes to the development of chronic liver disease. However, whether inflammation and complement activation occur in acute ERS and the relationship between complement activation and ERS and its effect on cell damage remain unclear. Therefore, ERS is a target for many diseases and possesses tremendous potential (King and Wilson, 2020). It is of great significance to study the effects of ERS on the body in order to explore the regulatory function of the liver.

Tunicamycin (TM) blocks protein glycosylation and induces ERS (Eo and Valentine, 2021). To date, *in vivo* animal models of ERS have mainly included rodents such as rats and mice. However, research has revealed considerable differences in RNA expression patterns between humans and mice, well beyond what was described previously, likely reflecting the fundamental physiological differences between these two organisms (Lin et al., 2014). Compared to rodents, pigs share a significant number of similarities with humans with regards to metabolism, susceptibility to various diseases (Jeong et al., 2019), and morphology and physiology of the liver (Lossi et al., 2016). Therefore, we chose to use TM to establish an ERS model in pigs to investigate the effects of ERS on liver injury, inflammatory reactions, and complement activation. Studies have shown that rats or mice intraperitoneally injected with 1 mg/kg body weight or 2 mg/kg body weight TM can be sacrificed 12, 24, or 48 h after injection to analyze ERS (Kim et al., 2016; Crider et al., 2018; Lee et al., 2018). Based on the equivalent dose conversion between these animals, piglets were sacrificed 48 h after intraperitoneal injection with 0.1 mg/kg or 0.3 mg/kg body weight TM in order to establish acute ERS models in this study. Recently, proteomics has become a cutting-edge method to study changes in total protein expression patterns and how the body responds to adverse effects (Ren et al., 2018). Proteomic analysis appears to reflect the phenotype more closely than genomic or transcriptomic studies, making it suitable for mechanistic studies and disease typing (Huang et al., 2020).

To further reveal the mechanism of liver injury caused by ERS and identify potential diagnostic biomarkers, the isobaric tags for relative and absolute quantitation (iTRAQ) technique was used to analyze the proteome of TM-induced ERS piglet livers as compared with the vehicle group. The parallel reaction monitoring (PRM) method was used to verify the differentially expressed proteins (DEPs) to confirm the reliability of the results. Bioinformatics was used to explore the specific proteins mobilized by ERS and the related signaling pathways regulated by them. The aim of this study was to investigate the effects of ERS on the liver, inflammation and the complement system and the molecular basis underlying the regulation of the body under ERS, which can provide a valuable resource for the treatment of ERS-related liver diseases.

## 2 Methods

### 2.1 Animal Ethics Statement

This study was conducted in the State Key Laboratory of Animal Nutrition. The experiments were conducted according to the guidelines of the Animal Welfare Committee of the Institute of Animal Science, Chinese Academy of Agricultural Science (IAS-CAAS; approval number: IAS-2018-20).

### 2.2 Animals and Establishment of the ERS Model

A total of twenty-one 35-day-old Duroc three-way crossbred barrows weighing 9.34 ± 0.3 kg were selected from seven litters and raised in an environmental control cabin maintained at 28°C and 55% humidity. After 1 week of environmental adaptation, to eliminate differences in the genetic background, three full siblings per litter were allocated to three groups as follows: 1) vehicle-treated group (5% dimethyl sulfoxide (DMSO), intraperitoneal injection); 2) TM-low dosage (LD)-treated group (intraperitoneal injection, 0.1 mg/kg body weight (bw)); and 3) TM-high dosage (HD)-treated group (intraperitoneal injection, 0.3 mg/kg bw). Each piglet was raised in a single cage, and seven piglets from the same treatment group were raised in one environmental control cabin. TM was dissolved in 5% DMSO and diluted with saline to obtain the desired concentration. All treated piglets were allowed free access to feed and drink.

The feed consumption of the piglets in each cage was recorded during the experiment, and the daily feed intake was calculated. Food intake was measured within 48 h after intraperitoneal injection. Jugular venous blood was collected from each pig via venipuncture using 10-ml BD Vacutainers (BD Biosciences, San Jose, CA, United States) containing sodium heparin. The collected blood was centrifuged at 3,000 × *g* for 15 min at 4°C. The supernatant plasma was collected and then stored in 1-ml Eppendorf tubes at −20°C until required.

Prior to the planned end of the experiment, euthanasia criteria were established using a head-only electric stun tong apparatus (Xingye Butchery Machinery Co. Ltd., Changde, Hunan Province, China) 48 h after the intraperitoneal injection of TM or vehicle. The liver was removed and weighed immediately, and the morphological images of the liver were obtained. The left lateral lobe of each liver was dissected, snap-frozen in liquid nitrogen, and stored at −80°C for mRNA and protein analyses. Additional liver samples were collected for paraffin embedding and immunohistochemical analyses.

### 2.3 Liver Morphological Examination

Fixed liver samples were prepared using conventional paraffin embedding techniques as described previously (Kim et al., 2018). Samples were sectioned at 5 µm thicknesses, mounted on slides, and stained with hematoxylin and eosin (H&E). Histological changes were investigated using a light microscope (Leica DM300, Wetzlar, Germany) under 200x magnification. The inflammation score of hepatic lobules was determined by counting the inflammatory lesions under a microscope at ×20 magnification as follows: 0 points for no lesions, 1 point for <2 lesions, 2 points for 2–4 lesions, and 3 points for >4 lesions (Li et al., 2022). The data were collected by an investigator who was blinded to the origin of the tissue sections.

### 2.4 Hepatic Triglyceride and Plasma Index Measurements

Pig liver tissue of the same quality was weighed, anhydrous ethanol was added, the tissue was fully ground, and the supernatant was collected after 3,000 r/min centrifugation. Hepatic triglyceride (TG) levels were measured using the relevant kits according to the manufacturer’s instructions (Sichuan Maccura Biotechnology, Sichuan, China). Hepatic TG measurements were performed using an automated chemistry analyzer (Hitachi 7600; Hitachi, Tokyo, Japan). Blood samples were collected in tubes containing sodium heparin and centrifuged to obtain plasma fractions. Plasma levels of aspartate aminotransferase (AST), alanine aminotransferase (ALT), TG, cholesterol (Chol), alkaline phosphatase (ALP), gamma-glutamyl transferase (GGT), total bilirubin (TBIL), and direct bilirubin (DBIL) were measured using relevant kits according to the manufacturer’s instructions (Sichuan Maccura Biotechnology). Levels of AST, ALT, TG, Chol, ALP, and GGT were measured using an automated chemistry analyzer (Hitachi 7600; Hitachi, Tokyo, Japan).

### 2.5 Measurements of Hepatic and Plasma Proinflammatory Cytokines

The levels of liver and plasma proinflammatory cytokines, including tumor necrosis factor alpha (TNF-α), C-reactive protein (CRP), and interleukin 6 (IL-6), were analyzed using enzyme-linked immunosorbent assay (ELISA) kits (R&D Systems, Minneapolis, MN, United States).

### 2.6 Plasma Complement Components Analysis

Colorimetry was used to detect the concentrations of complement factors C3 and C4, according to the manufacturer’s instructions (Huaying Bio-Tech Research Institute, Beijing, China). C5a levels were assessed using a commercial ELISA kit (Huaying, China). AP50 hemolytic complement activity was measured using rabbit blood cells and an ELISA kit (Huaying, China).

### 2.7 RT-qPCR Analysis

RNA isolation and RT-PCR were performed as previously described (Xin et al., 2018). Briefly, the same amount (15 mg) of each hepatic sample was homogenized in liquid nitrogen, and total RNA was isolated using the miRNeasy mini kit (Qiagen, Hilden, Germany). Complementary DNA (cDNA) synthesis was performed using reverse transcription with a PrimeScript RT reagent kit (TaKaRa, Dalian, China). Differences in gene expression were determined using qRT-PCR. Polymerase chain reaction quantification of each sample was performed in triplicate, and SYBR Green fluorescence (TaKaRa) was quantified using the CFX96 real-time system instrument (Bio-Rad, Hercules, CA, United States). Primer sequences for the ER stress-associated genes are listed in Supplementary Table S1. Relative expression levels were calculated using the 2^−ΔΔCt^ method, with β-actin as the reference.

### 2.8 Western Blot Analysis

Total protein was isolated from the frozen liver samples. Protein concentrations were measured using a bicinchoninic acid (BCA) assay (Beyotime Institute of Biotechnology, Shanghai, China). Hepatic protein samples were analyzed by Western blotting, as described previously (Xin et al., 2018). Protein samples were separated by electrophoresis on a 10% SDS-PAGE gel and transferred to a polyvinylidene difluoride membrane (Millipore, Billerica, MA, United States). The blotted membrane was incubated in 1× TBST blocking solution for 2 h at room temperature with 5% skimmed milk, washed, and then incubated with specific primary antibodies according to the experimental design overnight at 4°C. The following antibodies were used: anti-glucose-regulated protein 78 (GRP78) (1:1,000 dilution; SC-13968; Santa Cruz Biotechnology, Dallas, TX, United States), anti-glucose-regulated protein 94 (GRP94) (GRP94, 1:20,000 dilution; ab2791; Abcam, Cambridge, United Kingdom), anti-phosphorylated-IRE1 (p-IRE1) (1:4,000 dilution; NB100-2323; Novus Biologicals, Littleton, CO, United States), p-eukaryotic translation initiation factor 2α (p-eIF2α) (1:1,000 dilution; 3398; Cell Signaling Technology, Danvers, MA, United States), and β-actin (1:5,000 dilution; YM3028; Immunoway, Plano, TX, United States). The membranes were then washed in TBST and incubated with secondary antibodies (1:1,000 dilution; Invitrogen, Grand Island, NY, United States) for 1 h at room temperature. The blots were scanned using an Odyssey Infrared Imaging System (LI-COR Biosciences, Lincoln, NE, United States). Quantification of antigen-antibody complexes was performed using Quantity One analysis software (Bio-Rad).

### 2.9 Proteomics Sample

Pigs were stunned with electric shock rods 48 h after the intraperitoneal injection of TM or DMSO. The left lateral lobe of the liver was frozen in liquid nitrogen and preserved at −80°C. Four samples each were selected from the HD treatment group and the control group and stored at −80°C prior to iTRAQ detection and PRM protein verification. The experimental samples were divided into control group 1 and experimental group 2, with four biological repeats in each group, for a total of eight samples.

### 2.10 Protein Extraction

The sample was ground into a powder using liquid nitrogen. The powder was lysed in lysis buffer (2 M thiourea, 7 M urea, and 0.1% CHAPS, with protease inhibitor (Roche Applied Science, Indianapolis, IN, United States) and extracted thoroughly by ultrasonication (60 s, 0.2 s on, 2 s off, amplitude 22%). The stationary extract was then incubated for 30 min at room temperature, centrifuged at 15,000 × *g* for 1 h at 4°C, and the supernatant was collected. Finally, the protein concentration was determined by the Bradford assay using bovine serum albumin as the standard. Specific protein concentrations are listed in Supplementary Table S2.

### 2.11 Sodium Dodecyl Sulfate-Polyacrylamide Gel Electrophoresis (SDS-PAGE)

The protein profile of the extracted proteins was obtained by SDS-PAGE, according to the protocol described by Shang et al. (2019) with some modifications. Briefly, 20 µg of each protein sample in loading buffer was heated at 95°C for 5 min and then centrifuged at 25,000 × *g* for 5 min. The supernatant was loaded onto a 12% separating gel, which was then run at 120 V for 120 min. After staining with Coomassie brilliant blue solution R-250 for 2 h, the gel was placed in a destaining solution consisting of ethanol and acetic acid (4: 1, v/v) until the protein bands were clearly visible.

### 2.12 Filter-Aided Sample Preparation (FASP) Digestion

For digestion, the protein solution was reduced with 25 mM dithiothreitol for 60 min at 37°C, and alkylated with 50 mM iodoacetamide for 30 min at room temperature in the dark. The mixture was transferred to a 10 kDa ultrafiltration filter. Thereafter, the filter was washed three times with 300 μl of 20 mM triethylammonium bicarbonate buffer (TEAB), centrifuged at 12,000 × *g* for 10 min, washed using 300 μl of 50 mM TEAB, and centrifuged at 12,000 × *g* for 10 min. Finally, trypsin was added at a 1:50 trypsin: protein mass ratio for overnight digestion, washed three times with 300 μl of 50 mM TEAB, and then centrifuged at 12,000 × *g* for 10 min. SDS-PAGE gel images of the experimental samples are shown in Supplementary Figure 1.

### 2.13 iTRAQ Labeling

A 200-µg peptide mixture was labeled with iTRAQ reagents according to the manufacturer’s instructions (Applied Biosystems, Waltham, MA, United States). To label peptides with the iTRAQ reagent, 1 unit of label (defined as the amount of reagent required to label 100 μg of protein) was thawed and reconstituted in 150 μl of isopropanol.

### 2.14 High-pH Reversed-Phase (HpRP) Chromatography

After labeling, the samples were combined into one tube and dried in vacuum. Dried peptides were resuspended in 100 μl of mobile phase A and centrifuged at 14,000 × *g* for 20 min. The supernatants were loaded onto a column (Durashell-C18, 4.6 × 250 mm, 5 μm, 100 Å, Agela, DC952505-0) and eluted stepwise by injecting mobile phase B into a RIGOL L-3000 system (RIGOL, Beijing, China). Mobile phase A consisted of 2% (v/v) acetonitrile and 98% (v/v) ddH_2_O at pH 10, and phase B consisted of 98% (v/v) acetonitrile and 2% (v/v) ddH_2_O at pH 10. The 60 min gradients comprised 5% mobile phase B for 5 min, 5–30% mobile phase B for 35 min, 30–95% mobile phase B for 10 min, and equilibration with 5% mobile phase B for 10 min at a 300 nl/min flow rate. Fractions were eluted at 1.5 min intervals and collected using the step gradients of mobile phase B.

### 2.15 LC–MS/MS Analysis

#### 2.15.1 For iTRAQ

LC–MS/MS analysis was conducted using an Orbitrap Fusion Lumos mass spectrometer (Thermo Fisher Scientific, Waltham, MA, United States) combined with an EASY-nLC 1000 nanosystem (Thermo Fisher Scientific, Waltham, MA, United States). The peptide mixtures were separated using a binary solvent system with 99.9% H_2_O, 0.1% formic acid (phase A), and 80% acetonitrile, 0.1% formic acid (phase B). Linear gradients consisted of 4–38% B for 90 min, 38–56% B for 20 min, 56–100% B for 6 min, and 100% B for 4 min at a flow rate of 600mnl/min. The eluent was submitted to an Orbitrap Fusion Lumos MS system. The applied electrospray voltage was 2.0 kV. The full scan MS mode was operated with the following parameters: automatic gain control (AGC) target, 5e5; resolution, 12,000 FWHM; scan range, 350–1,550 m/z; maximum injection time, 50 ms; and collision energy, 35%. The MS/MS mode was set as follows: AGC target, 5e4; resolution, 15,000 FWHM; and maximum injection time, 22 ms.

#### 2.15.2 For PRM

The peptides were re-dissolved in 10 μl 0.1% formic acid (FA), and 1 ul of each sample was used to make a mixed sample. Each sample of one microgram was analyzed using a self-made analytical column (75 μm × 100 mm, 3 μm) on an EASY-nLC1200 connected to a Q Exactive Plus mass spectrometer (Thermo Fisher Scientific, Waltham, MA, United States). Peptides were eluted using a binary solvent system with 99.9% H_2_O, 0.1% formic acid (phase A), and 80% acetonitrile, 19.9% H_2_O, and 0.1% formic acid (phase B). The following linear gradient was used: 6–12% B for 2 min, 12–28% B for 36 min, 28–38% B for 11 min, 38%–100% B for 6 min, and 100% B for 5 min at a flow rate of 0.6 μl/min. The eluent was introduced directly to a Q-Exactive Plus mass spectrometer via an EASY-Spray ion source. The source ionization parameters were as follows: spray voltage, 2.3 kV; capillary temperature, 320°C.

For the DDA MS runs, one full scan MS from 350 to 1,600 *m*/*z* followed by 20 MS/MS scans were continuously acquired. The resolution for MS was set to 70,000, and that for MS/MS was set to 17,500. For high-energy collision dissociation (HCD), the isolation window was set to 2 *m*/*z* and a normalized collision energy of 30% was applied. Dynamic exclusion was applied 20 s after the second fragmentation event.

A total of 20 proteins were selected for PRM verification. A target peptide list consisting of 39 peptides was determined from the selected proteins based on the iTRAQ experiment. In the MS method, one full scan MS from 350 to 1,150 *m*/*z*, followed by 39 targeted scans were acquired. The AGC target was 3e6 and the maximum injection time was 20 ms. The resolution for MS was set to 70,000, and that for MS/MS was set to 17,500. For the HCD, the isolation window was set to 1.6 *m*/*z* and a normalized collision energy of 27% was applied.

### 2.16 Database Search

#### 2.16.1 Mascot (for iTRAQ)

ProteoWizard (version 3.0.8789) was used to extract the raw data files from the MS. The MS/MS samples were searched using the Mascot search engine against the Sus scrofa database (UniProt). The search was governed by the following parameters: trypsin was assumed as the digestion enzyme; two missed cleavages were allowed at maximum; a fragment ion mass tolerance of 0.02 Da was used; a parent ion tolerance of 10 ppm was used; carbamidomethylation of cysteine was selected as a fixed modification; and oxidation of methionine, iTRAQ 8 plex of lysine, and the N-terminus were set as variable modifications. Scaffold (version Scaffold_4.6.2, Proteome Software Inc., Portland, OR, United States) was used to validate MS/MS-based peptide and protein identifications. Peptide identifications were accepted if they could be established with greater than 90.0% probability to achieve an FDR of less than 1.0% using the Scaffold Local FDR algorithm. Protein identifications were accepted if they could be established at greater than 99.0% probability to achieve an FDR of less than 1.0% and contained at least two identified peptides. Protein probabilities were assigned using the Protein Prophet algorithm (Nesvizhskii, Al et al. Anal. Chem. 2003; 75 (17):464658). To ensure protein reliability, all quantified proteins have at least two unique peptides.

#### 2.16.2 Skyline (for PRM)

PRM data were set up and searched using Skyline (version 4.1.1.11725) software. The database construction data were DDA data and the unique peptide with the highest value or the top two quantitative values of the target protein export were selected to set the PRM method. For the PRM data, an NCBI database (Sus_refesq_20180716. Fasta) was selected as the background, mixed DDA data were selected as the library, and the top eight product ions in the library were used for comparison and quantification; the data were deemed reliable when the peak shape was intact and the retention time was within the set retention time range, undetected product ions were manually removed, and the target polypeptide quantitative value was output.

### 2.17 Statistical Analysis

All statistical analyses were performed using SAS version 9.4 (SAS Institute, Inc., Cary, NC, United States). Differences between means were assessed using one-way analysis of variance (ANOVA), followed by Duncan’s test for multiple comparisons. Data are presented as mean ± SD, and statistical significance was set at *p* < 0.05. The protein quantitative data of the two groups of samples being compared were tested by the group *t*-test, and the proteins with *p*-value ≤ 0.05 were screened. The selection of differential candidate proteins must be judged based on multiple changes and statistical significance. In the data analysis, proteins with a ratio ≤ (1 − 2 × SD) or ≥ (1 + 2 × SD) and *p*-value ≤ 0.05 were selected as candidate differential proteins. The Pearson correlation analysis between the plasma proinflammatory cytokines, plasma complement factors, liver proinflammatory cytokines, and the DEPs in the complement and coagulation cascade pathway was performed.

## 3 Results

### 3.1 Establishment of an ERS Model in Piglets

**FIGURE 1:**
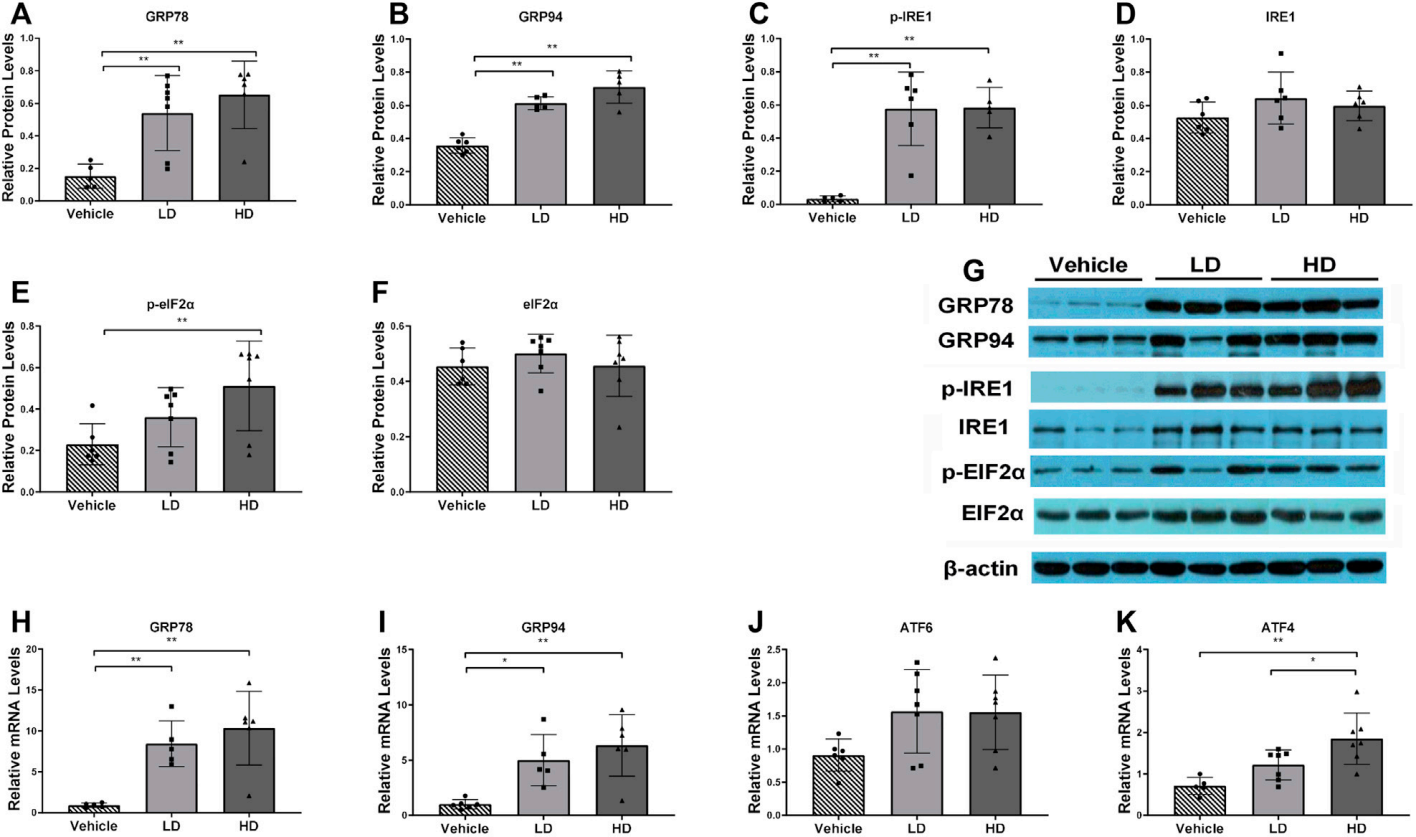
ERS model was successfully established in piglets using different doses of tunicamycin. Piglets were administered with low dosage (LD) tunicamycin (TM) (0.1 mg/kg body weight), high dosage (HD) TM (0.3 mg/kg body weight), or vehicle (5% DMSO) for 48 h. **(A–F)** Relative protein levels of GRP94, GRP78, p-IRE1, IRE1, p-EIF2α, EIF2α, and β-actin were assessed using Western blot analysis in different TM treatment groups. **(H–K)** Relative mRNA levels of GRP94, GRP78, ATF6, and ATF4 were measured by real-time RT-PCR. * means *p* < 0.05; ** means *p* < 0.01.

To evaluate ERS development and detect the expression levels of hepatic ERS-associated genes and proteins in piglets, we performed RT-PCR and Western blot analyses. Protein expression levels of GRP78, GRP94, p-IRE1, and were estimated in liver samples, as shown in Figures 1 A–D. GRP78, GRP94, and IRE1α protein levels were significantly increased (*p* < 0.01) in both the LD and HD groups compared with those in the vehicle control group, and the p-EIF2α protein expression level was significantly increased (*p* < 0.01) in the HD group compared with that in the vehicle group. The activation of ERS-linked genes, including GRP78, GRP94, ATF4, and ATF6, is shown in Figures 1 F–I. We found that, compared with levels in the control group, the gene expression level of GRP78 was significantly upregulated (*p* < 0.01) in both the HD and LD groups, the gene expression level of GRP94 was significantly upregulated in both the HD (*p* < 0.01) and LD (*p* < 0.05) groups, the gene expression level of ATF4 was significantly increased (*p* < 0.01) in the HD group, and the gene expression level of ATF6 was not significantly different. Our data indicate that low and high TM doses stimulated the development of ERS and UPR in piglets.

### 3.2 ERS-Dependent Changes in Piglets’ Physiological Traits

**FIGURE 2:**
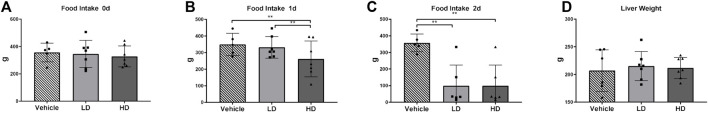
TM-induced ERS effects on physiological traits of piglets. Piglets were administered with TM LD (0.1 mg/kg body weight), TM HD (0.3 mg/kg body weight), or vehicle for 48 h. **(A–C)** Food intake in different groups on day0, day1 and day2. **(D)** Liver weight gain in different groups. * means *p* < 0.05; ** means *p* < 0.01.

We monitored changes in food intake and liver weight in piglets within 48 h of intraperitoneal injections of TM (or vehicle) (Figure 2). TM/HD-treated piglets demonstrated decreased food intake within 24 h compared to the vehicle control group (*p* < 0.01), while TM/LD-treated piglets showed stable parameters. However, 24 h later, food intake drastically declined in piglets in the LD and HD groups (63 and 67%, respectively). Notably, no differences were found between the extracted liver weights of the TM-treated and control animals.

### 3.3 Liver Damage Under ERS

**FIGURE 3:**
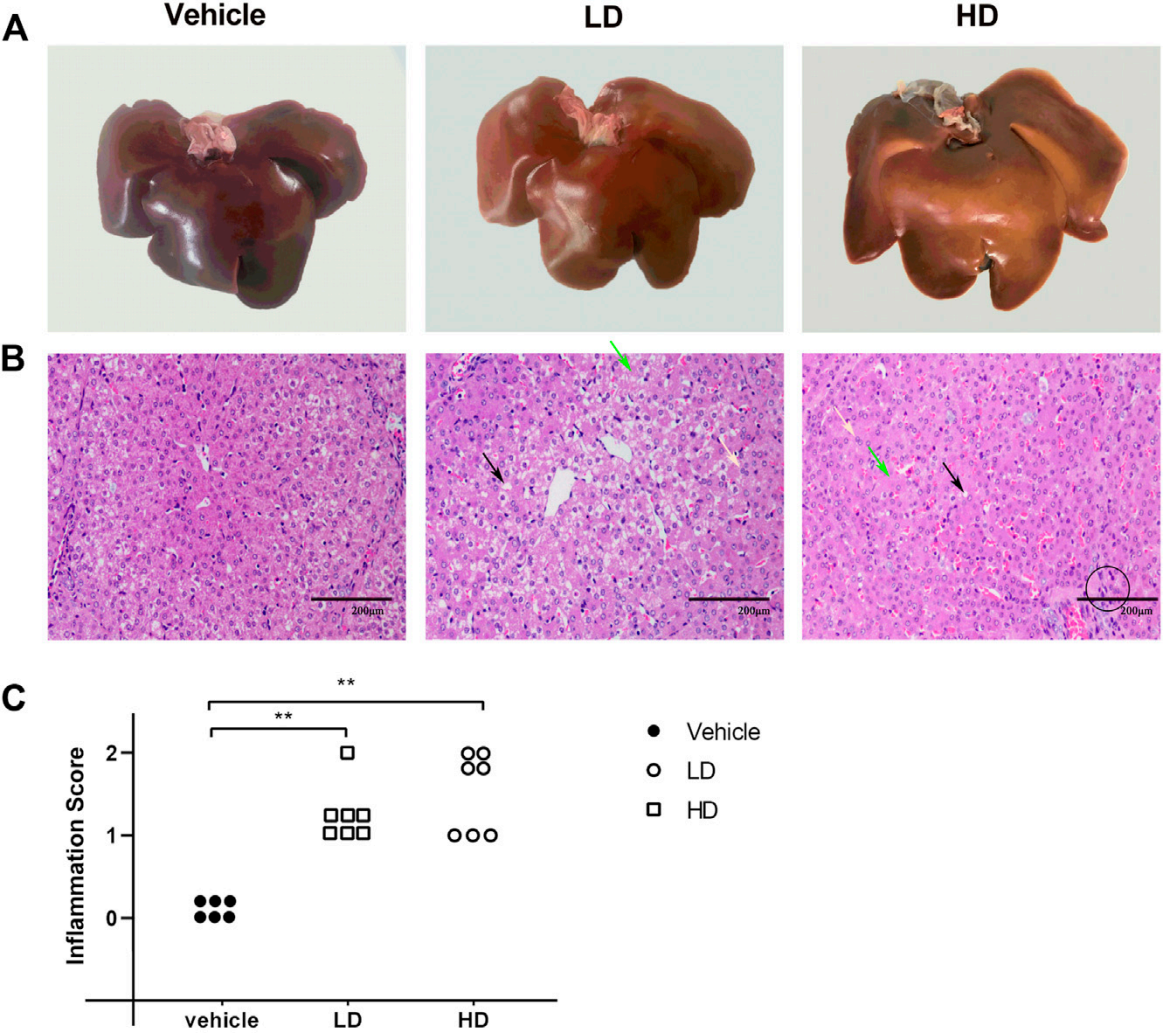
TM-induced ERS causes liver injury in piglets. Piglets were administered with TM LD (0.1 mg/kg body weight), HD (0.3 mg/kg body weight), or vehicle for 48 h. **(A)** Liver morphology in different groups. **(B)** H&E staining of liver tissues in different groups. The black arrow denotes lipid droplets in the liver, green arrow denotes hydropic degeneration in hepatocytes, and yellow arrow denotes dual-core. The black circle denotes the accumulation of inflammatory cells as the lesion of the inflammation score. **(C)** Inflammation score of hepatic lobules in different groups. * means *p* < 0.05; ** means *p* < 0.01.

H&E analysis, liver morphologies, and inflammation scores of hepatic lobules are shown in Figures 3 A–C. A yellowish tissue color in the liver was observed after TM treatment, especially in the TM/HD group. H&E staining revealed that, in the control group, the structure of the liver lobules was clear and complete, and the liver cells were arranged radically centered in the central vein. However, upon exposure to TM, an increased binuclear phenomenon, accumulation of lipid droplets, and diffuse hydropic degeneration were observed. The inflammation score of hepatic lobules was determined by counting the inflammatory lesions of the liver tissue under a 20× microscope. The results showed that the inflammation score of both LD and HD groups was significantly higher (*p* < 0.01) than that of the vehicle group, and the inflammation score revealed that compared with the control group both LD and HD groups had inflammatory reactions.

### 3.4 TM/ERS-Dependent Changes in Liver Function

**FIGURE 4:**
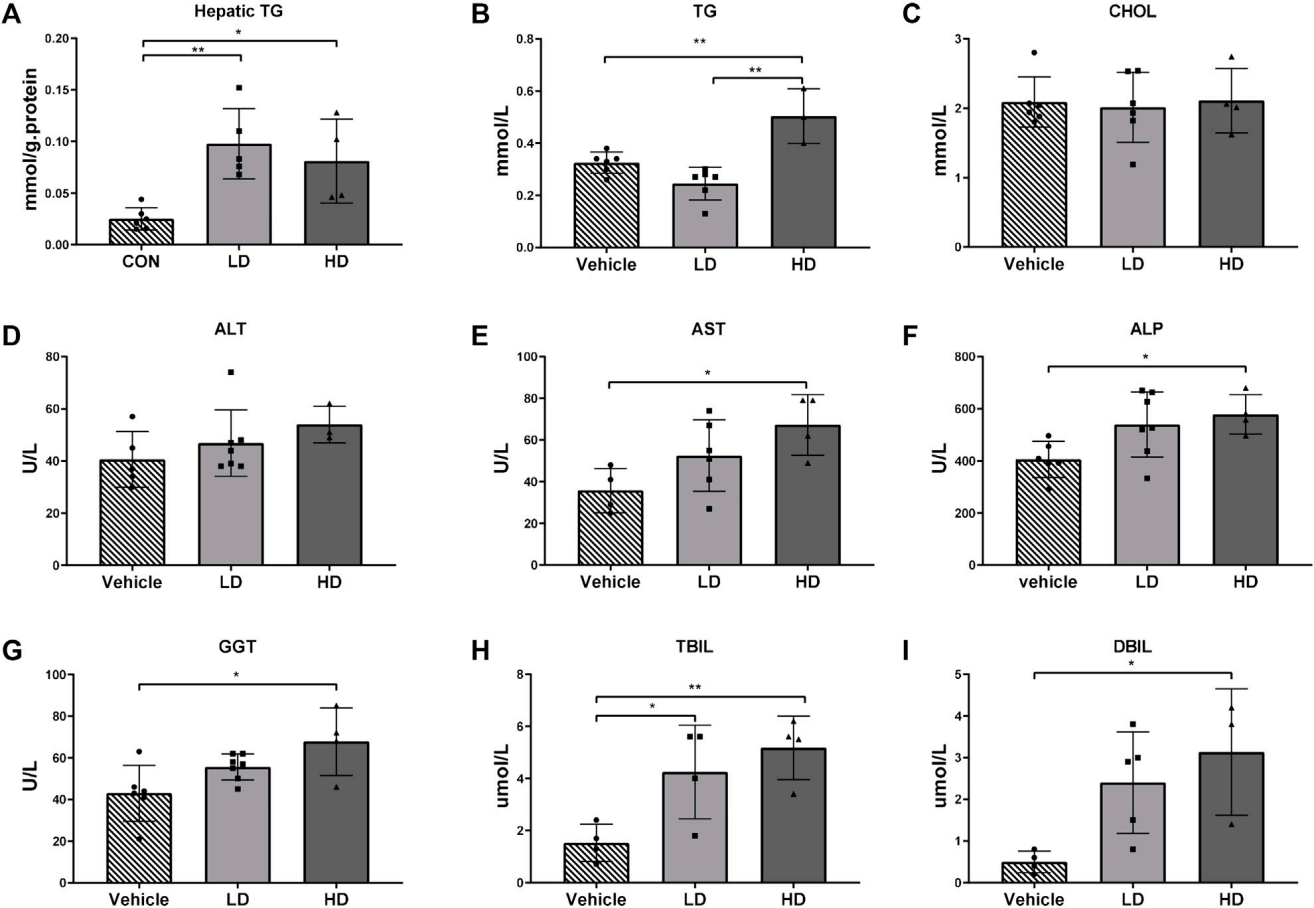
TM-induced ERS impairs liver function in piglets. Piglets were administered with TM LD (0.1 mg/kg body weight), HD (0.3 mg/kg body weight), or vehicle for 48 h. **(A)** Hepatic TG levels in different groups. **(B–I)** Plasma level of TG, CHOL, ALT, AST, ALP, GGT, TBIL, and DBIL in different groups. * means *p* < 0.05; ** means *p* < 0.01.

The hepatic TG level was significantly increased in both the LD (*p* < 0.01) and HD groups (*p* < 0.05), indicating an increased hepatic lipid accumulation following TM treatment, which shows that TM-induced ERS can promote liver steatosis. Plasma TG and CHOL levels were analyzed as indicators of altered lipid metabolism. Plasma AST, ALT, ALP, GGT, TBIL, and DBIL levels were analyzed as indicators of altered liver function. Plasma TG levels were significantly higher (*p* < 0.01) in the HD group than in the LD and vehicle control groups, demonstrating that lipid metabolism was disturbed in the HD group. The plasma levels of AST, ALP, and GGT were significantly higher (*p* < 0.05) in the HD group than in the vehicle control group, demonstrating that TM-activated ERS caused significant liver damage. Plasma levels of TBIL were significantly higher in the HD (*p* < 0.01) and LD groups (*p* < 0.01), and plasma levels of DBIL were significantly higher in the HD group than those in the vehicle control group, demonstrating that both LD and HD had disorders of bilirubin metabolism, which indicates the occurrence of jaundice. The results are shown in Figure 4.

### 3.5 ERS-Stimulated Changes in Levels of Proinflammatory Cytokines

**FIGURE 5:**
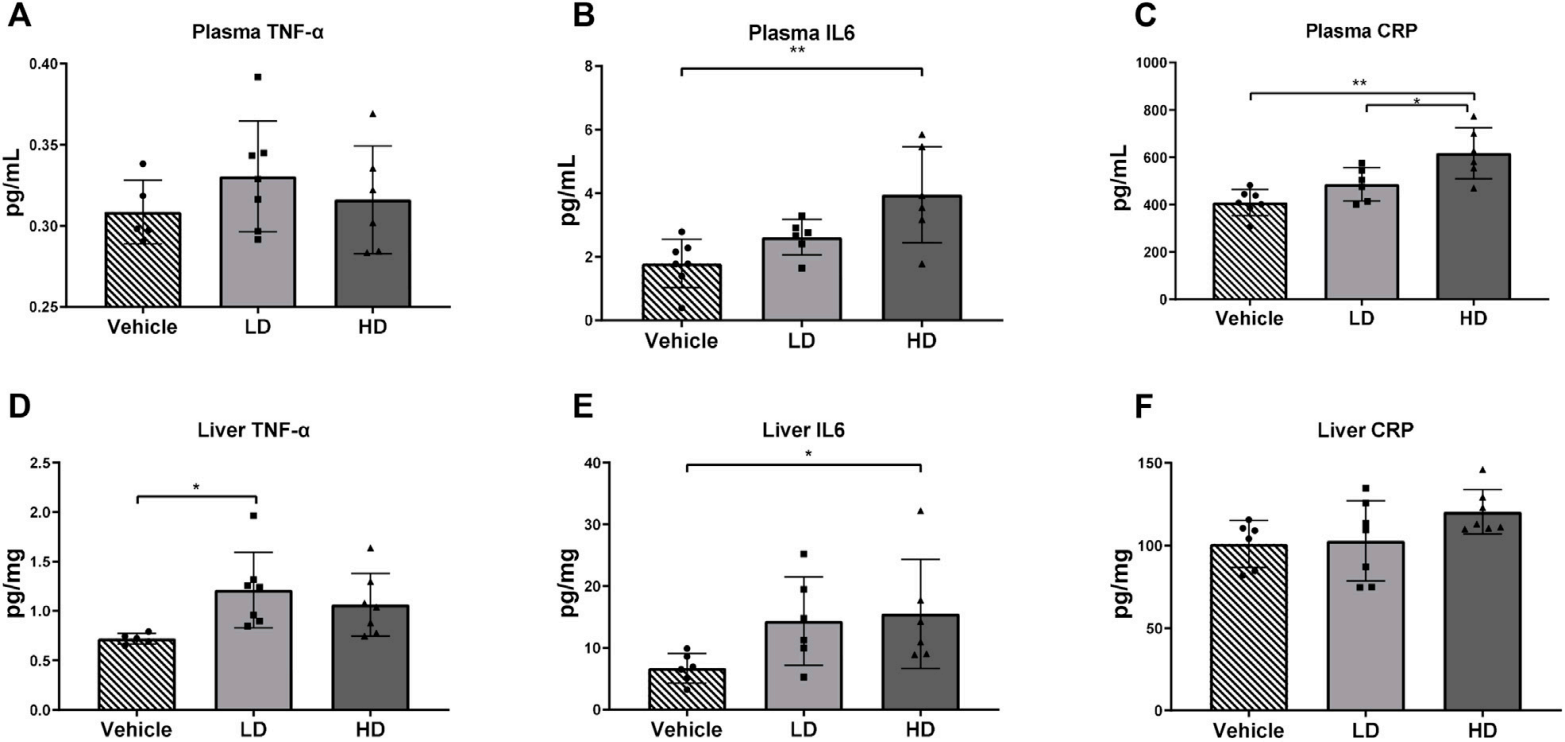
TM-induced ERS triggers the inflammatory reaction in piglets. Piglets were administered with TM LD (0.1 mg/kg body weight), HD (0.3 mg/kg body weight), or vehicle for 48 h. **(A–C)** Plasma level of TNF-α, IL-6, and CRP in different groups. **(D–F)** Hepatic level of TNF-α, IL-6, and CRP in different groups. * means *p* < 0.05; ** means *p* < 0.01.

Our data showed that the HD group piglets had significantly higher plasma CRP levels compared with those in the vehicle group (*p* < 0.01) and the LD group (*p* < 0.05). Both plasma and liver tissue levels of IL-6 were elevated in piglets in the HD group treated with TM. The TNF-α concentration in the liver samples of the LD group was significantly higher than that in the vehicle group (*p* < 0.05); however, plasma TNF-α was not affected by TM treatment. The results are shown in Figure 5.

### 3.6 Complement Activation Under ERS

**FIGURE 6:**
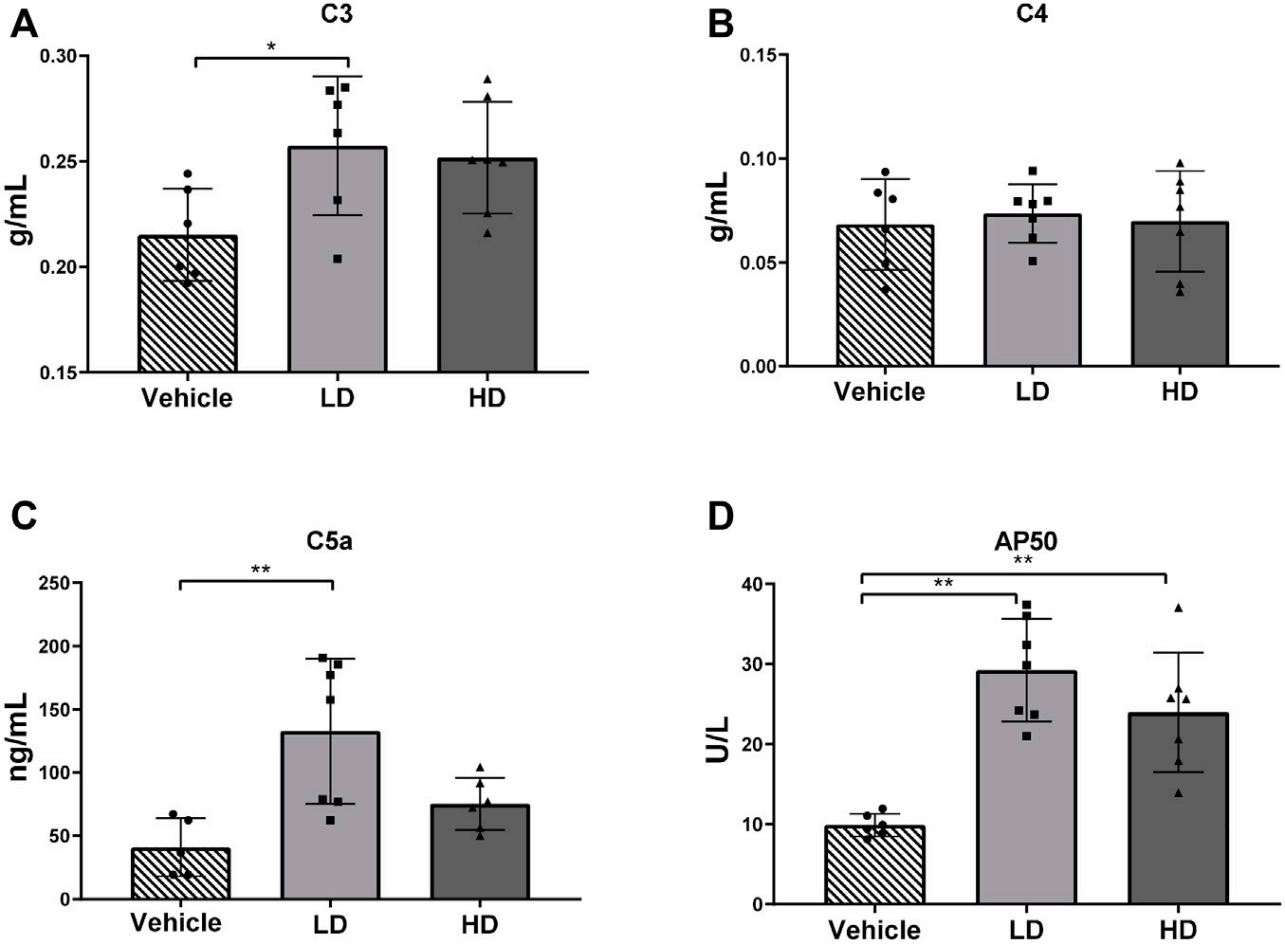
TM-induced ERS triggers complement activation in piglets. Piglets were administered with TM LD (0.1 mg/kg body weight), HD (0.3 mg/kg body weight), or vehicle for 48 h. **(A–D)** Plasma level of C3, C4, C5a, and AP50 in different groups. * means *p* < 0.05; ** means *p* < 0.01.

To determine ERS-associated changes in activation of the complement system, plasma complement factors C3, C4, C5a, and AP50 were measured as indicated in Figures 6 A–D. Plasma concentrations of complement factors C3 and C5a increased in the LD group, and the plasma concentration of AP50 increased significantly (*p* < 0.01) in both the LD and HD groups compared with that in the control group. However, plasma C4 levels were not significantly different than those in the controls.

### 3.7 Identification and Quantification of Differentially Abundant Proteins

**FIGURE 7:**
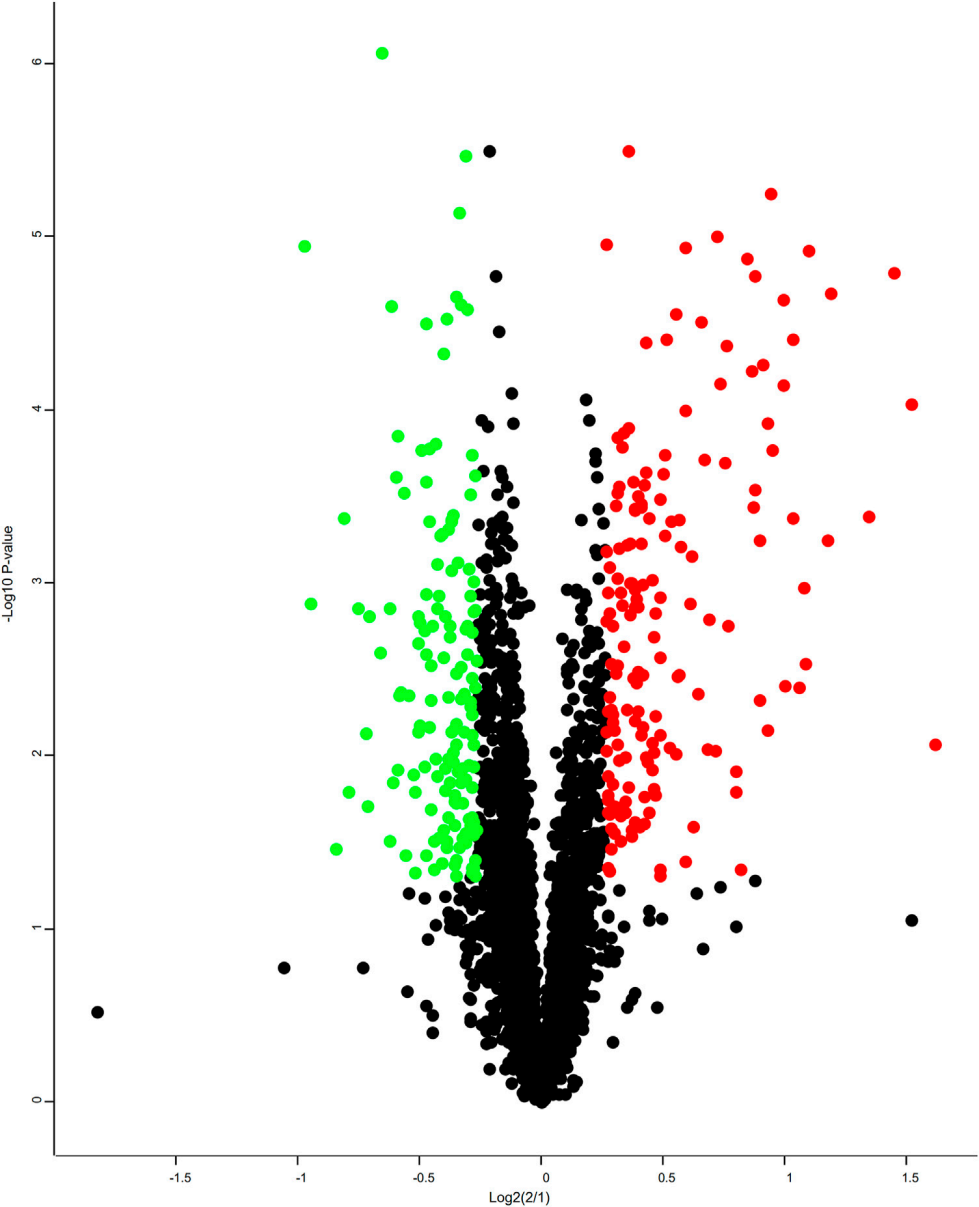
Volcano plots. Each point in the differentially expressed volcanic map represents a protein, the abscissa is the log2 logarithm of the protein ratio between the two groups, and the ordinate is the –log10 logarithm of the *p*-value between the two groups. The greater the abscissa absolute value, the greater the difference in protein expression between the two groups of samples; the greater the vertical coordinate value, the more significant the differential expression, and the more reliable the screened DEPs. The green dots represent downregulated DEPs, the red dots represent upregulated DEPs, and the gray dots represent non-differentially expressed proteins.

A total of 3,679 proteins were identified by the iTRAQ analysis (Supplementary Table S3), all of which were co-quantitative proteins. In this experiment, the candidate proteins were screened according to multiple changes (Folder > 1.2 or < 0.83 and *p* < 0.05), and 311 DEPs were screened (Supplementary Tables S4–S6). Among them, 146 proteins were downregulated and 165 proteins were upregulated. A volcano plot was constructed to visualize differences in protein expression levels between the two groups of samples, as shown in Figure 7.

### 3.8 Bioinformatics Analysis

**FIGURE 8:**
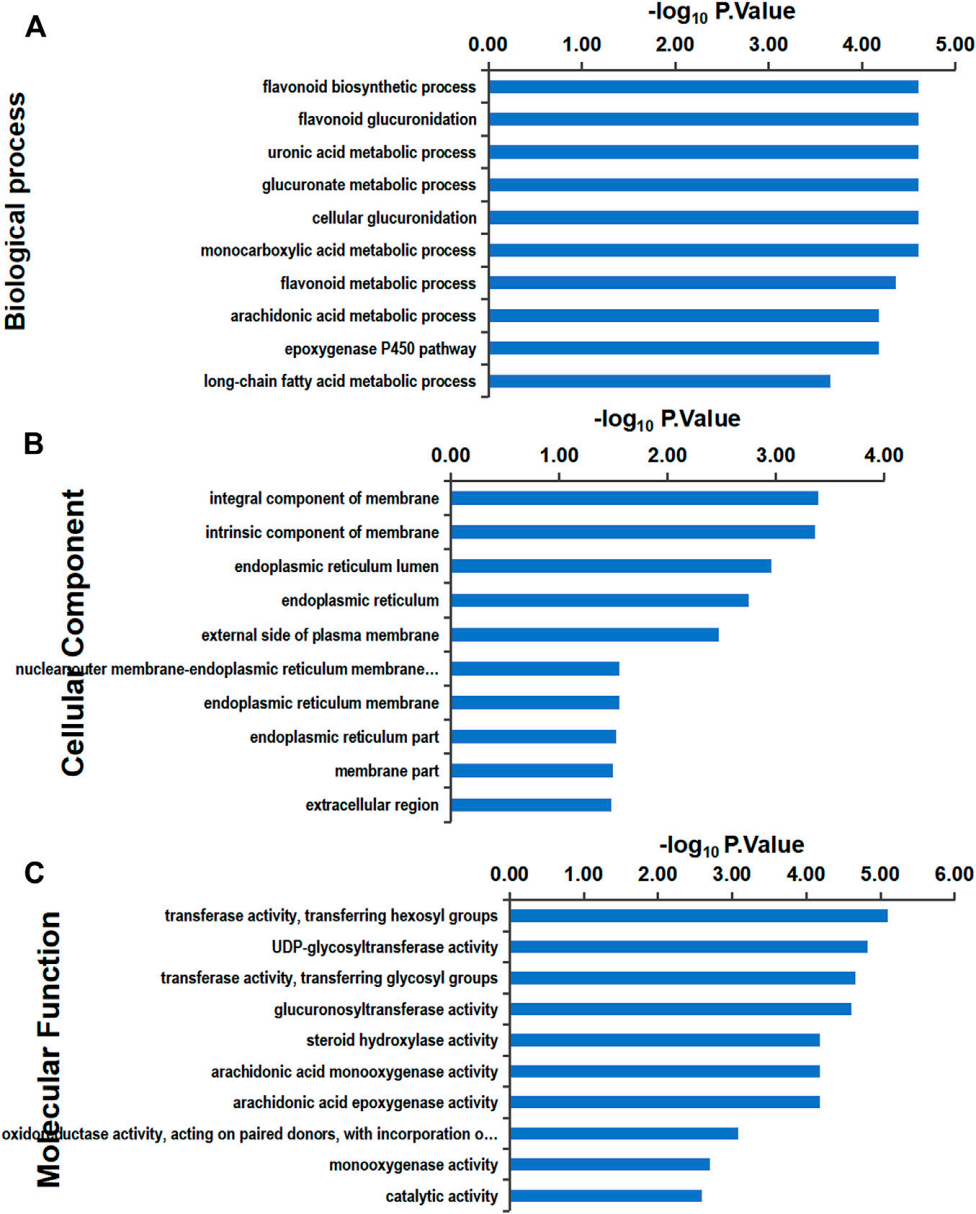
GO enrichment analysis: the ordinate is the GO item, and the abscissa represents the enrichment of differential proteins in corresponding functional items. The larger the value of –log10 *p*-value is, the more relevant the differential proteins are to the function, and the analysis of differential proteins with this function can be focused on. Gene Ontology (GO) functional classification **(A)** GO annotation in terms of cellular components. **(B)** GO annotation in terms of molecular function. **(C)** GO annotation in terms of biological processes.

In this study, the Gene Ontology (GO) signal pathway enrichment analysis of DEPs was performed using Blast2GO software, which found that the main enriched biological processes were flavonoid metabolism and uronic acid metabolism, both of which function in liver detoxification. Additional biological processes that were identified include arachidonic acid metabolism, which is involved in inflammatory reactions and immune system regulation, and nutrient metabolism, which is mainly enriched in fatty acid metabolism. The top 10 clusters of biological processes, cell components, and molecular functions are shown in Figure 8.

**FIGURE 9:**
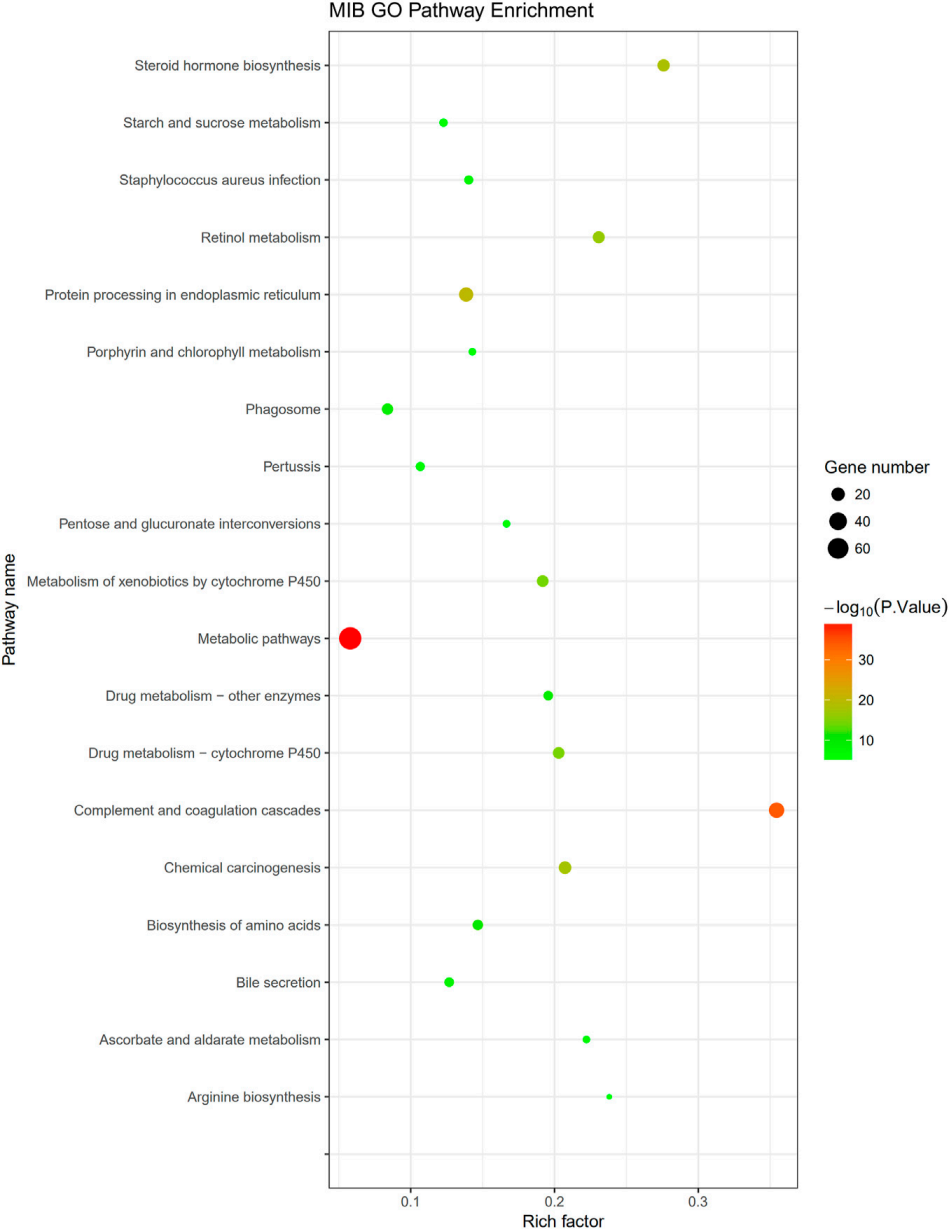
Pathway enrichment: the vertical axis represents the name of the pathway, and the horizontal axis represents the rich factor (the ratio of the number of DEPs enriched in the pathway to the number of annotated proteins). The greater the enrichment factor, the more significant the enrichment level of DEPs in this pathway. Each dot in the figure represents a KEGG pathway, and the size of the dot represents the number of proteins enriched in the pathway. The smaller the *p*-value, the more reliable the enrichment significance of DEPs in this pathway.

**FIGURE 10:**
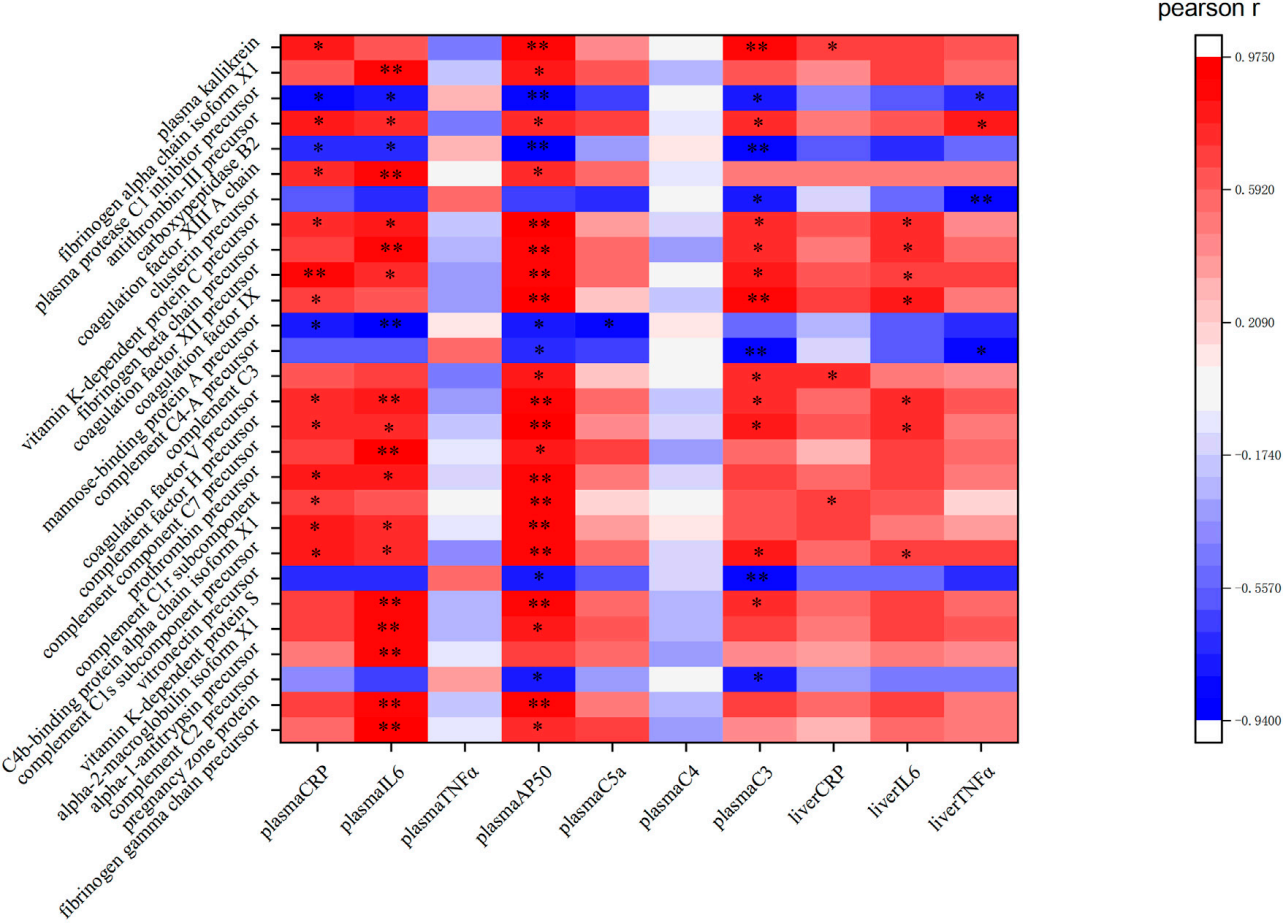
Pearson correlation analysis between the proinflammatory cytokines, plasma complement factors, and the DEPs in the complement and coagulation cascade pathway: red represents a positive correlation, while green represents a negative correlation. ** and * in the figure mean the values of *p* < 0.01 and *p* < 0.05, respectively.

The KEGG pathway analysis performed in this study indicated 311 DEPs that were enriched for 209 signaling pathways. The main enriched signaling pathways were the metabolic signaling pathway (47 downregulated DEPs and 25 upregulated DEPs), the complement and coagulation cascade pathway (5 downregulated DEPs and 28 upregulated DEPs), and processing in the endoplasmic reticulum (19 upregulated DEPs and 4 downregulated DEPs), which indicates that TM treatment altered metabolism and abnormally activated the complement and coagulation cascade pathways and the endoplasmic reticulum protein processing pathway. DEPs were labeled in the pathway map in order to visualize their distribution, and maps of the complement and coagulation cascade pathways and protein processing in the endoplasmic pathway are shown in Supplementary Figures S2, S3. The top 20 signaling pathways identified in the KEGG analysis are shown in Figure 9. Pearson correlation analysis (Figure 10) showed that most of the DEPs in the complement and coagulation pathways were significantly correlated with plasma CRP, plasma IL6, and plasma AP50. This result demonstrated that plasma proinflammatory factors and complement factors participated in the activation of the complement and coagulation cascade pathways in the liver under ERS.

### 3.9 PRM Protein Verification

**FIGURE 11:**
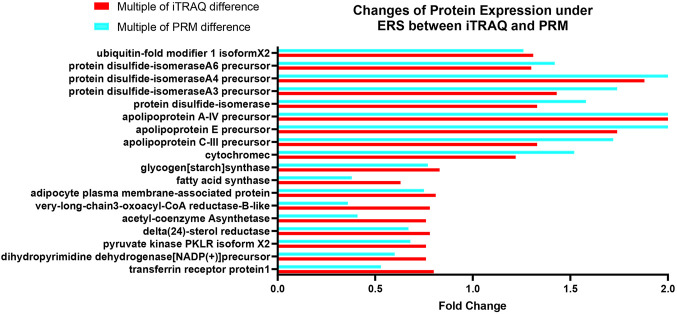
Changes of protein expression under ERS between iTRAQ and PRM bar chart: the horizontal coordinate is the proteins involved in PRM verification, and the vertical coordinate is the fold change of the protein.

Both iTRAQ and PRM quantify proteins using liquid chromatography and tandem mass spectrometry (LC–MS). To further verify the proteins identified by iTRAQ, 18 of the 311 DEPs were selected for PRM verification, including proteins involved in lipid metabolism, endoplasmic reticulum, and redox reactions, which are closely related to the main KEGG pathway. The expression trends of the 18 DEPs were the same in PRM as those in iTRAQ (Figure 11), indicating that the results of iTRAQ in this experiment were reliable for further analysis.

### 3.10 Differentially Expressed Proteins Interaction Analysis

**FIGURE 12:**
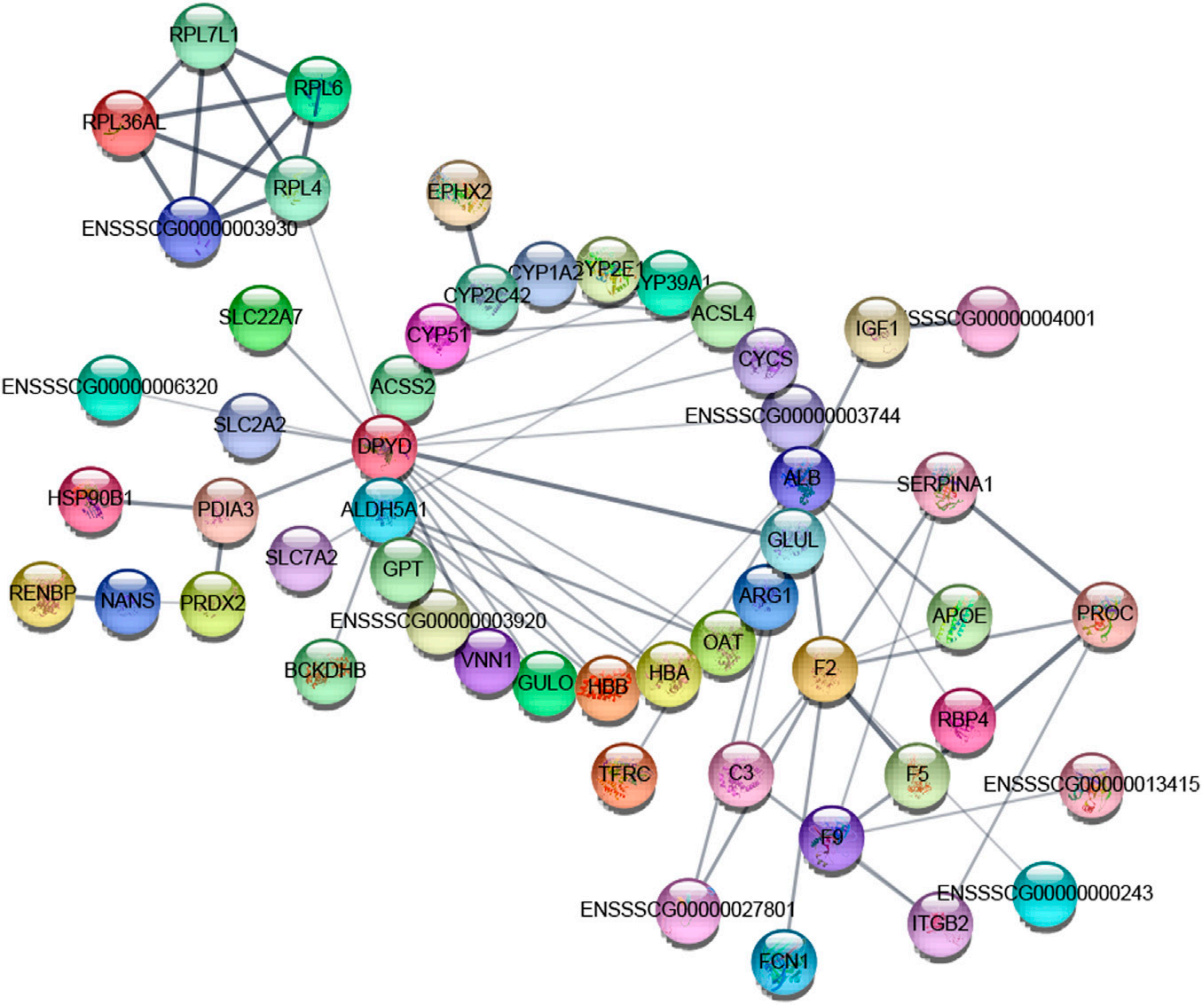
Interaction of differentially expressed proteins: the node in the graph is a protein, and the edge is interaction. The thickness of the edge in the interaction network indicates the strength of the interaction between the two nodes connected by the edge, and there is no known interaction between the unconnected protein nodes.

In this experiment, the DEP information was compared with porcine species proteins found in the STRING database to obtain the protein interaction information, and a network map of DEP interactions was constructed using the String online analysis site. The map is shown in Figure 12.

## 4 Discussion

### 4.1 Effects of ERS on Liver Injury and Inflammatory Reactions

Most *in vivo* research models of ERS use mice, rats, and guinea pigs. TM is an internationally recognized ERS inducer that blocks N-linked glycosylation and causes cell cycle impediments *in vivo* and *in vitro*. Under normal physiological conditions, three proximal UPR sensors associate with GRP78; during ERS, GRP78 binds misfolded proteins and releases PERK, ATF6, and IRE1 to relieve the ER pressure. Activated PERK phosphorylates the alpha subunit of EIf2α (p-EIF2α), which in turn attenuates global translation initiation and protein synthesis. IRE1α, which is the most highly conserved UPR sensor, restores the protein folding capacity and ER associated-protein degradation (ERAD), which relieves the ER pressure. The current study showed that a single injection of 0.1 or 0.3 mg/kg TM elevated ERS markers in the livers of treated piglets, indicating that ERS model pigs were established for the first time.

ERS is related to the development of liver diseases such as NAFLD (Flessa et al., 2021). In this study, ERS and liver damage were detected in TM-treated pigs. We identified lipid droplet accumulation and inflammation in the liver sections of the LD and HD groups. In addition, plasma TG levels were significantly increased in the HD group compared with those in the LD and vehicle groups, verifying that a high dose of TM caused lipid disturbance. Moreover, the hepatic TG levels were significantly higher in the LD and HD groups than in the control group, indicating steatosis in the livers of the LD and HD groups. Following TM treatment, the color of the liver was yellowish, implying the presence of jaundice. Bilirubin is a vital basis for the clinical diagnosis of jaundice because dysfunction of the hepatobiliary system leads to an unhealthy buildup of bilirubin in the blood, resulting in jaundice (Jacob et al., 2020). In this study, TBIL increased significantly in both the LD and HD groups, indicating the occurrence of jaundice, and DBIL and GGT levels increased significantly in the HD group, illustrating the occurrence of liver jaundice. Plasma ALT levels had a tendency to increase, and the AST and ALP levels increased significantly in the HD group compared to those in the control group. Increased ALP levels occurring with jaundice indicate liver hepatitis and liver damage. In this study, TM injections led to jaundice, steatosis, and abnormal liver function. Adverse effects were prominent in piglets exposed to higher TM doses. Additionally, the food intake of TM-treated piglets decreased sharply, which may have been associated with TM-induced metabolic dysfunction and hepatic steatosis (Chen et al., 2021).

In total, three distinct UPR branches are involved in the activation of NF-κB, which regulates the production of proinflammatory cytokines such as IL-6 and TNF-α. ATF4, an ERS marker protein, regulates the production of inflammatory signaling molecules (Robinson et al., 2016). Levels of both IL-6 and TNF-α, upstream effectors of CRP production in the liver, have been shown to increase in the livers of mice under ERS conditions (Kim et al., 2014). CRP is a nonspecific biomarker of inflammation and an acute phase protein that increases when the body is infected and returns to a normal level after homeostasis is maintained (Gessner et al., 2015). In this study, levels of IL-6 and TNF-α in the liver and IL-6 and CRP in the plasma increased in TM-treated piglets, indicating that 48 h of TM treatment induced acute ERS. In conclusion, acute ERS stimulates inflammation in piglets.

Complements are components of the innate and adaptive immune systems. ERS acts on the complement coagulation cascade in two ways. On the one hand, when cells or tissues are under ERS, the molecular chaperone protein GRP78 is upregulated to initiate the coagulation cascade (Pozza and Austin, 2005), and C3, the central component of the complement system, is co-secreted with the ERS marker protein endoplasmin, which may facilitate C3 cleavage (Chaumonnot et al., 2021), and then anaphylatoxin C3a and C5a can trigger the inflammatory reaction. On the other hand, ERS causes pathological damage to cells and tissues and mediates subsequent coagulation cascades. Activation of the classical complement pathways (C3 and C4) and the alternative complement pathway (AP50) were detected in the current study. TM treatment increased the levels of plasma AP50 and C3 in piglets, suggesting that activation of the complement system was accompanied by liver injury. C5a, a component of the terminal complement complex (C5b-9), could further intensify inflammatory responses and elevate the production of proinflammatory cytokines, including TNF-α and IL-6. CRP, another biomarker of inflammatory responses, activates the classical complement pathway in damaged tissues (Korkmaz et al., 2017). Regulatory relationships between complement activation (C3 and AP50) and inflammatory markers (CRP, IL-6, and TNF-α), which were responsible for the observed liver injury in this study, remain to be clarified.

### 4.2 GO Analysis

Proteomic analysis was performed to further investigate the potential mechanism of ERS effects on piglet livers in the HD and vehicle groups. GO enrichment analysis of DEPs showed that they were mainly involved in the biological processes of flavonoid biosynthesis and uronic acid metabolism, both of which function in liver detoxification, indicating the toxicity of TM. Some studies have found that TM can be used as a clinically powerful treatment for some cancers (Gu et al., 2021); therefore, when using TM in medicine, attention should be paid to protecting the liver. The protein cysteine dioxygenase type 1 from the biological process category of cellular glucuronidation (GO:0032787) was also found to be upregulated (ratio = 1.6600). It is the rate-limiting enzyme involved in taurine biosynthesis, and taurine has antioxidant effects and ameliorates ERS and NAFLD (Gentile et al., 2011; Batista et al., 2013; Hagiwara et al., 2014), indicating that piglet livers may respond to ERS by increasing taurine levels. The downregulated hepatokine protein fetuin B (ratio = 0.7025) from the extracellular region in the cellular component category (GO:0005576) can impair insulin action in hepatocytes (Wang et al., 2018), and previous studies have indicated that the administration of fetuin B aggravated hepatic lipid accumulation *in vitro* and *in vivo* (Zhou et al., 2019). The decreased fetuin B level in this study indicates that suppressing fetuin B may be an important mechanism for coping with ERS. Levels of the protein Arginase-2 (ARG-2) from catalytic activation in the molecular function category (GO:0003824) were found to be increased (ratio = 2.7271). ARG-2 deficiency leads to spontaneous development of hepatic steatosis and proinflammatory activation (Navarro et al., 2015). The increased ARG-2 level indicates that activation of the catalytic function of ARG-2 may be involved in the ERS response.

### 4.3 DEPs Involved in Protein Processing in the Endoplasmic Reticulum

In our study, an ERS piglet model was successfully established, and in this pathway, 19 DEPs were upregulated and four DEPs were downregulated, indicating that the pathway of protein processing in the endoplasmic reticulum was activated. Ubiquitin thioesterase OTU1, which is involved in the UPR and participates in the ubiquitin-related ERAD process (Gaudet et al., 2011), was significantly increased in this study (ratio = 1.2102), indicating the enhancement of ERAD to ameliorate ER pressure. Hypoxia upregulated protein 1, also known as the 150 kDa oxygen-regulated protein (ORP150), has a pivotal cytoprotective function in hypoxia-induced cellular perturbation (Ozawa et al., 1999). A study used a small interfering RNA to show that kynurenic acid ameliorates ERS and liver steatosis through ORP150 signaling (Pyun et al., 2021). In this study, ORP150 levels were increased (ratio = 2.14128), indicating that it may play an important role in coping with ERS and steatosis. DnaJ homolog subfamily C member 10 (DNAJC10) was upregulated in this study (ratio = 1.4031). This protein promotes the correct folding and degradation of misfolded proteins but also promotes the intrinsic apoptotic signaling pathway in response to ERS (Ushioda et al., 2008), which may be one of the reasons why the UPR effects both survival and apoptosis.

### 4.4 DEPs Involved in Lipid Metabolism

In the lipid metabolism pathway, 47 DEPs were downregulated, and 25 DEPs were upregulated, indicating that this pathway was altered. In fatty liver disease, increased ALT2 may be the cause of the increase in ALT activity in mice with steatosis (Jadhao et al., 2004); therefore, ALT2 effectively reflects liver function. In this study, the increased ALT2 level (ratio = 1.92998) verified that the TM injection caused liver damage. Fat in the liver is transported out in the form of very low density lipoproteins (VLDL), supplied as energy in the form of β-oxidation, or deposited in the liver in the form of TG. Fatty acid synthase (FAS) is responsible for the *de novo* synthesis of new fatty acids. In this study, FAS was significantly downregulated (ratio = 0.632). Many enzymes downregulated in this study were associated with fat synthesis, indicating that liver lipid synthesis is inhibited under TM injection, which may be due to the acute ERS-affected ER structure that is responsible for the synthesis of lipids. It may also be due to the negative feedback of increased liver TG accumulation during adipogenesis in the HD group (Giudetti et al., 2021). Levels of most of the apolipoproteins were changed in this study, which was verified by PRM. Apolipoprotein A-IV (ApoA-IV, ratio = 3.07) promotes the expansion of VLDL to alleviate the lipid burden of the liver (VerHague et al., 2013; Wang et al., 2015). ApoA-IV was significantly increased in this study, indicating that it may exert positive effects on VLDL for lipid transport out of the liver to relieve ERS. In liver microsomes, cytochrome P450 2C42 (CYP2C42) is involved in the NADPH-dependent electron transport pathway, which oxidizes a variety of compounds, including steroids, fatty acids, and xenobiotics (Gaudet et al., 2011). In this experiment, increased CYP2C42 (ratio = 1.7033) indicated that augmented fatty acid oxidation is part of the ERS response mechanism.

### 4.5 DEPs Involved in the Complement Coagulation Cascade Pathway

In the complement coagulation cascade pathway, five DEPs were downregulated and 28 DEPs were upregulated. Most of the DEPs in this pathway were correlated with the plasma cytokines and complement factors, indicating that this pathway was activated. Plasma protease C1 inhibitor is responsible for the negative regulation of complement activation (Matsushita et al., 2000). In correlation analysis, this protein was also negatively correlated with CRP, IL6, AP50, and C3 in plasma and TNFα in the liver, suggesting the importance of this protein in mediating inflammation and the complement system. In this study, its precursor was decreased (ratio = 0.7553), indicating a weakening of its inhibition of the complement system. The protein fibrinogen beta chain (ratio = 1.68235) and fibrinogen alpha chain (ratio = 1.2696) were significantly increased in our experiment, which are involved in the cellular response of IL-6 (Ljungman et al., 2009). Correlation analysis also showed that both of them were negatively correlated with plasma IL6 significantly (*p* < 0.01), and both of them mediate endothelial cell survival and have an anti-apoptotic effect (Pluskota and D'Souza, 2000). Therefore, these proteins may protect the liver through anti-apoptotic effects and are regulated by inflammation under TM treatment. In our study, mannose-binding protein A, an initiator of the leptin pathway of complement activation, was significantly decreased (ratio = 0.7572) and negatively correlated with plasma CRP, IL6, AP50, and C5a. Recently, it has been found that activation of the lectin pathway might be involved in systemic worsening of the inflammatory response (Niederreiter et al., 2022), and the depletion of this protein exhibits strong protection from ischemia/reperfusion injury (Neglia et al., 2020), so the downregulated mannose-binding protein A may be involved in the liver protection under inflammatory response and complement activation. Vitamin K-dependent protein C is activated by thrombin (ratio of prothrombin = 1.5880), which inhibits apoptosis and inflammatory reactions but plays a positive role in protecting the endothelial cell barrier (Ding et al., 2015). Furthermore, the vitamin K-dependent protein C not only correlated with plasma inflammatory cytokines and complement factors but also positively correlated with IL-6 expression in the liver, indicating this protein may play an important role in liver inflammation. The increase in vitamin K-dependent protein C (ratio = 1.3377) indicates the protection of complement activation and the complex relationship between complement and inflammation. Carboxypeptidase B2 (ratio = 0.7266) can function as an anaphylatoxin inhibitor (Zhou et al., 2022). Correlation analysis also showed that this protein was negatively correlated with plasma C3 and AP50, indicating that in the early phase of ERS, the body will downregulate this protein to assure the subsequent complement system activation. In drug-induced liver injury, the loss of anaphylatoxin, such as C3a, leads to the obstruction of liver regeneration (Markiewski et al., 2004); thus, activation of the complement system can promote liver regeneration after liver injury, and an increase in complement cytokines can increase the cellular ubiquitination level, thereby promoting the elimination of unfolded proteins to maintain homeostasis (Kitzler et al., 2012). Therefore, activation of the complement coagulation cascade pathway may be responsible for protecting cells under ERS.

## 5 Conclusion

In this study, we used TM to establish a piglet ERS model and found that ERS could cause liver steatosis, liver damage, and complement system activation accompanied by inflammation. A total of 311 DEPs were screened using iTRAQ analysis, and GO analysis showed that DEPs were mainly enriched in detoxification reactions. KEGG analysis suggested that most DEPs were associated with metabolic pathways, complement and coagulation cascade pathways, and protein processes in the ER pathway. Under ERS, the protein folding ability of the ER and the apoptosis signal were regulated, fat synthesis-related enzymes were downregulated, and proteins involved in fatty acid oxidation and transportation were enhanced to ameliorate liver steatosis. In the complement system, a large number of complement factors and anti-apoptosis factors were increased, which play an essential role in protecting the liver from damage. Correlation analysis showed that most DEPs in the complement and coagulation cascade pathway were closely related to plasma proinflammatory factors and complement factors. These results provide valuable information for the adaptive mechanisms of piglet livers under ERS and could help identify vital functional genes to apply as possible diagnostic biomarkers and treatment for diseases induced by ERS in the future.

## Data Availability

The datasets presented in this study can be found in online repositories. The names of the repository/repositories and accession number(s) can be found in the article/Supplementary Material.

## References

[B1] Batista T. M. da Silva P. M. R. Amaral A. G. Ribeiro R. A. Boschero A. C. Carneiro E. M. (2013). Taurine Supplementation Restores Insulin Secretion and Reduces ER Stress Markers in Protein-Malnourished Mice. Adv. Exp. Med. Biol. 776, 129–139. 10.1007/978-1-4614-6093-0_14 23392878

[B2] Chaumonnot K. Masson S. Sikner H. Bouchard A. Baverel V. Bellaye P.-S. (2021). The HSP GRP94 Interacts with Macrophage Intracellular Complement C3 and Impacts M2 Profile during ER Stress. Cell Death Dis 12 (1), 114. 10.1038/s41419-020-03288-x 33483465 PMC7822929

[B3] Chen Q. Fang W. Cui K. Chen Q. Xiang X. Zhang J. (2021). Endoplasmic Reticulum Stress Induces Hepatic Steatosis by Transcriptional Upregulating Lipid Droplet Protein Perilipin2. FASEB J. 35 (10), e21900. 10.1096/fj.202100739RR 34547130

[B4] Crider A. Nelson T. Davis T. Fagan K. Vaibhav K. Luo M. (2018). Estrogen Receptor β Agonist Attenuates Endoplasmic Reticulum Stress-Induced Changes in Social Behavior and Brain Connectivity in Mice. Mol. Neurobiol. 55 (9), 7606–7618. 10.1007/s12035-018-0929-8 29430617 PMC6070416

[B5] Ding Q. Yang L. Dinarvand P. Wang X. Rezaie A. R. (2015). Protein C Thr315Ala Variant Results in Gain of Function but Manifests as Type II Deficiency in Diagnostic Assays. Blood 125 (15), 2428–2434. 10.1182/blood-2014-12-617274 25651845 PMC4392011

[B6] Eo H. Valentine R. J. (2021). Imoxin Inhibits Tunicamycin-Induced Endoplasmic Reticulum Stress and Restores Insulin Signaling in C2C12 Myotubes. Am. J. Physiol. Cel Physiol 321, C221–C229. 10.1152/ajpcell.00544.2020 34077277

[B7] Flessa C.-M. Kyrou I. Nasiri-Ansari N. Kaltsas G. Kassi E. Randeva H. S. (2021). Endoplasmic Reticulum Stress and Autophagy in the Pathogenesis of Non-Alcoholic Fatty Liver Disease (NAFLD): Current Evidence and Perspectives. Curr. Obes. Rep. 10, 134–161. 10.1007/s13679-021-00431-3 33751456

[B8] Gardner B. M. Pincus D. Gotthardt K. Gallagher C. M. Walter P. (2013). Endoplasmic Reticulum Stress Sensing in the Unfolded Protein Response. Cold Spring Harbor Perspect. Biol. 5 (3), a013169. 10.1101/cshperspect.a013169 PMC3578356 23388626

[B9] Gaudet P. Livstone M. S. Lewis S. E. Thomas P. D. (2011). Phylogenetic-Based Propagation of Functional Annotations within the Gene Ontology Consortium. Brief. Bioinform. 12 (5), 449–462. 10.1093/bib/bbr042 21873635 PMC3178059

[B10] Gentile C. L. Nivala A. M. Gonzales J. C. Pfaffenbach K. T. Wang D. Wei Y. (2011). Experimental Evidence for Therapeutic Potential of Taurine in the Treatment of Nonalcoholic Fatty Liver Disease. Am. J. Physiol. Regul. Integr. Comp. Physiol. 301 (6), R1710–R1722. 10.1152/ajpregu.00677.2010 21957160 PMC3233850

[B11] Gessner D. K. Gröne B. Couturier A. Rosenbaum S. Hillen S. Becker S. (2015). Dietary Fish Oil Inhibits Pro-Inflammatory and ER Stress Signalling Pathways in the Liver of Sows during Lactation. PLoS One 10 (9), e0137684. 10.1371/journal.pone.0137684 26351857 PMC4564272

[B12] Giudetti A. M. Vergara D. Longo S. Friuli M. Eramo B. Tacconi S. (2021). Oleoylethanolamide Reduces Hepatic Oxidative Stress and Endoplasmic Reticulum Stress in High-Fat Diet-Fed Rats. Antioxidants 10 (8), 1289. 10.3390/antiox10081289 34439537 PMC8389293

[B13] Gu C. Zhang Y. Chen D. Liu H. Mi K. (2021). Tunicamycin-induced Endoplasmic Reticulum Stress Inhibits Chemoresistance of FaDu Hypopharyngeal Carcinoma Cells in 3D Collagen I Cultures and *In Vivo* . Exp. Cel Res. 405 (2), 112725. 10.1016/j.yexcr.2021.112725 34224701

[B14] Hagiwara A. Ishizaki S. Takehana K. Fujitani S. Sonaka I. Satsu H. (2014). Branched-Chain Amino Acids Inhibit the TGF-Beta-Induced Down-Regulation of Taurine Biosynthetic Enzyme Cysteine Dioxygenase in HepG2 Cells. Amino Acids 46 (5), 1275–1283. 10.1007/s00726-014-1693-3 24553827 PMC3984414

[B15] Huang D. Wang Y. Lv J. Yan Y. Hu Y. Liu C. (2020). Proteomic Profiling Analysis of Postmenopausal Osteoporosis and Osteopenia Identifies Potential Proteins Associated with Low Bone mineral Density. PeerJ 8 (7), e9009. 10.7717/peerj.9009 32328356 PMC7164430

[B16] Jacob S. S. Bankapur A. Barkur S. Acharya M. Chidangil S. Rao P. (2020). Micro-Raman Spectroscopy Analysis of Optically Trapped Erythrocytes in Jaundice. Front. Physiol. 11, 821. 10.3389/fphys.2020.00821 32754052 PMC7366392

[B17] Jadaho S. B. Yang R.-Z. Lin Q. Hu H. Anania F. A. Shuldiner A. R. (2004). Murine Alanine Aminotransferase: cDNA Cloning, Functional Expression, and Differential Gene Regulation in Mouse Fatty Liver. Hepatology 39 (5), 1297–1302. 10.1002/hep.20182 15122758

[B18] Jeong P.-S. Yoon S.-B. Lee M.-H. Son H.-C. Lee H.-Y. Lee S. (2019). Embryo Aggregation Regulates *In Vitro* Stress Conditions to Promote Developmental Competence in Pigs. PeerJ 7, e8143. 10.7717/peerj.8143 31844571 PMC6913270

[B19] Kim M.-H. Aydemir T. B. Cousins R. J. (2016). Dietary Zinc Regulates Apoptosis through the Phosphorylated Eukaryotic Initiation Factor 2α/Activating Transcription Factor-4/C/EBP-Homologous Protein Pathway during Pharmacologically Induced Endoplasmic Reticulum Stress in Livers of Mice. J. Nutr. 146 (11), 2180–2186. 10.3945/jn.116.237495 27605406 PMC5086795

[B20] Kim S. Joe Y. Jeong S. O. Zheng M. Back S. H. Park S. W. (2014). Endoplasmic Reticulum Stress Is Sufficient for the Induction of IL-1β Production via Activation of the NF-κB and Inflammasome Pathways. Innate Immun. 20 (8), 799–815. 10.1177/1753425913508593 24217221

[B21] Kim S. Kwon D.-y. Kwak J.-H. Lee S. Lee Y.-H. Yun J. (2018). Tunicamycin-Induced ER Stress Is Accompanied with Oxidative Stress via Abrogation of Sulfur Amino Acids Metabolism in the Liver. Int. J. Mol. Sci. 19 (12), 4114. 10.3390/ijms19124114 PMC6321199 30567393

[B22] King A. P. Wilson J. J. (2020). Endoplasmic Reticulum Stress: An Arising Target for Metal-Based Anticancer Agents. Chem. Soc. Rev. 49 (22), 8113–8136. 10.1039/d0cs00259c 32597908

[B23] Kitzler T. M. Papillon J. Guillemette J. Wing S. S. Cybulsky A. V. (2012). Complement Modulates the Function of the Ubiquitin-Proteasome System and Endoplasmic Reticulum-Associated Degradation in Glomerular Epithelial Cells. Biochim. Biophys. Acta 1823 (5), 1007–1016. 10.1016/j.bbamcr.2012.03.001 22426620

[B24] Korkmaz H. I. Krijnen P. A. J. Ulrich M. M. W. de Jong E. van Zuijlen P. P. M. Niessen H. W. M. (2017). The Role of Complement in the Acute Phase Response after burns. Burns 43 (7), 1390–1399. 10.1016/j.burns.2017.03.007 28410933

[B25] Lee M.-M. Kim H.-G. Lee S.-B. Lee J.-S. Kim W.-Y. Choi S.-H. (2018). CGplus, a Standardized Herbal Composition Ameliorates Non-alcoholic Steatohepatitis in a Tunicamycin-Induced Mouse Model. Phytomedicine 41, 24–32. 10.1016/j.phymed.2018.01.020 29519316

[B26] Li L. Xu M. He C. Wang H. Hu Q. (2022). Polystyrene Nanoplastics Potentiate the Development of Hepatic Fibrosis in High Fat Diet Fed Mice. Environ. Toxicol. 37 (2), 362–372. 10.1002/tox.23404 34755918

[B27] Li Y.-M. Zhao S.-Y. Zhao H.-H. Wang B.-H. Li S.-M. (2021). Procyanidin B2 Alleviates Palmitic Acid-Induced Injury in HepG2 Cells via Endoplasmic Reticulum Stress Pathway. Evidence-Based Complement. Altern. Med. 2021, 8920757. 10.1155/2021/8920757 PMC8702323 34956386

[B28] Lin S. Lin Y. Nery J. R. Urich M. A. Breschi A. Davis C. A. (2014). Comparison of the Transcriptional Landscapes between Human and Mouse Tissues. Proc. Natl. Acad. Sci. U.S.A. 111 (48), 17224–17229. 10.1073/pnas.1413624111 25413365 PMC4260565

[B29] Ljungman P. Bellander T. Schneider A. Breitner S. Forastiere F. Hampel R. (2009). Modification of the Interleukin-6 Response to Air Pollution by Interleukin-6 and Fibrinogen Polymorphisms. Environ. Health Perspect. 117 (9), 1373–1379. 10.1289/ehp.0800370 19750100 PMC2737012

[B30] Lossi L. D’Angelo L. De Girolamo P. Merighi A. (2016). Anatomical Features for an Adequate Choice of Experimental Animal Model in Biomedicine: II. Small Laboratory Rodents, Rabbit, and Pig. Ann. Anat. - Anatomischer Anzeiger 204, 11–28. 10.1016/j.aanat.2015.10.002 26527557

[B31] Markiewski M. M. Mastellos D. Tudoran R. DeAngelis R. A. Strey C. W. Franchini S. (2004). C3a and C3b Activation Products of the Third Component of Complement (C3) Are Critical for Normal Liver Recovery after Toxic Injury. J. Immunol. 173 (2), 747–754. 10.4049/jimmunol.173.2.747 15240660

[B32] Matsushita M. Thiel S. Jensenius J. C. Terai I. Fujita T. (2000). Proteolytic Activities of Two Types of Mannose-Binding Lectin-Associated Serine Protease. J. Immunol. 165 (5), 2637–2642. 10.4049/jimmunol.165.5.2637 10946292

[B33] McCullough R. L. McMullen M. R. Sheehan M. M. Poulsen K. L. Roychowdhury S. Chiang D. J. (2018). Complement Factor D Protects Mice from Ethanol-Induced Inflammation and Liver Injury. Am. J. Physiology-Gastrointestinal Liver Physiol. 315 (1), G66–g79. 10.1152/ajpgi.00334.2017 PMC6109707 29597356

[B34] Navarro L. A. Wree A. Povero D. Berk M. P. Eguchi A. Ghosh S. (2015). Arginase 2 Deficiency Results in Spontaneous Steatohepatitis: A Novel Link between Innate Immune Activation and Hepatic De Novo Lipogenesis. J. Hepatol. 62 (2), 412–420. 10.1016/j.jhep.2014.09.015 25234945 PMC4736721

[B35] Neglia L. Oggioni M. Mercurio D. De Simoni M.-G. Fumagalli S. (2020). Specific Contribution of Mannose-Binding Lectin Murine Isoforms to Brain Ischemia/Reperfusion Injury. Cell Mol Immunol 17 (3), 218–226. 10.1038/s41423-019-0225-1 30967639 PMC7052250

[B36] Niederreiter J. Eck C. Ries T. Hartmann A. Märkl B. Büttner-Herold M. (2022). Complement Activation via the Lectin and Alternative Pathway in Patients with Severe COVID-19. Front. Immunol. 13, 835156. 10.3389/fimmu.2022.835156 35237273 PMC8884149

[B37] Ozawa K. Kuwabara K. Tamatani M. Takatsuji K. Tsukamoto Y. Kaneda S. (1999). 150-kDa Oxygen-Regulated Protein (ORP150) Suppresses Hypoxia-Induced Apoptotic Cell Death. J. Biol. Chem. 274 (10), 6397–6404. 10.1074/jbc.274.10.6397 10037731

[B38] Pluskota E. D'Souza S. E. (2000). Fibrinogen Interactions with ICAM-1 (CD54) Regulate Endothelial Cell Survival. Eur. J. Biochem. 267 (15), 4693–4704. 10.1046/j.1432-1327.2000.01520.x 10903502

[B39] Pozza L. M. Austin R. C. (2005). Getting a GRP on Tissue Factor Activation. Arterioscler Thromb. Vasc. Biol. 25 (8), 1529–1531. 10.1161/01.ATV.0000177041.47444.e2 16055754

[B40] Puri P. Mirshahi F. Cheung O. Natarajan R. Maher J. W. Kellum J. M. (2008). Activation and Dysregulation of the Unfolded Protein Response in Nonalcoholic Fatty Liver Disease. Gastroenterology 134 (2), 568–576. 10.1053/j.gastro.2007.10.039 18082745

[B41] Pyun D. H. Kim T. J. Kim M. J. Hong S. A. Abd El‐Aty A. M. Jeong J. H. (2021). Endogenous Metabolite, Kynurenic Acid, Attenuates Nonalcoholic Fatty Liver Disease via AMPK/Autophagy‐ and AMPK/ORP150‐Mediated Signaling. J. Cel Physiol 236 (7), 4902–4912. 10.1002/jcp.30199 33283879

[B42] Ren B. Wang Y. Wang H. Wu Y. Li J. Tian J. (2018). Comparative Proteomics Reveals the Neurotoxicity Mechanism of ER Stressors Tunicamycin and Dithiothreitol. Neurotoxicology 68, 25–37. 10.1016/j.neuro.2018.07.004 30003905

[B43] Robinson M. W. Cathal H. Cliona O. F. (2016). Liver Immunology and its Role in Inflammation and Homeostasis. Cell Mol. Immunol. 13 (3), 267–276. 10.1038/cmi.2016.3 27063467 PMC4856809

[B44] Rutkowski D. T. Wu J. Back S.-H. Callaghan M. U. Ferris S. P. Iqbal J. (2008). UPR Pathways Combine to Prevent Hepatic Steatosis Caused by ER Stress-Mediated Suppression of Transcriptional Master Regulators. Develop. Cel 15 (6), 829–840. 10.1016/j.devcel.2008.10.015 PMC2923556 19081072

[B45] Shang W.-H. Han J.-R. Yan J.-N. Du Y.-N. Xu Y.-S. Xue C.-F. (2019). Quantitative Proteome Reveals Variation in the Condition Factor of Sea Urchin Strongylocentrotus Nudus during the Fishing Season Using an iTRAQ-Based Approach. Mar. Drugs 17 (7), 397. 10.3390/md17070397 PMC6669438 31284417

[B46] Ushioda R. Hoseki J. Araki K. Jansen G. Thomas D. Y. Nagata K. (2008). ERdj5 Is Required as a Disulfide Reductase for Degradation of Misfolded Proteins in the ER. Science 321 (5888), 569–572. 10.1126/science.1159293 18653895

[B47] VerHague M. A. Cheng D. Weinberg R. B. Shelness G. S. (2013). Apolipoprotein A-IV Expression in Mouse Liver Enhances Triglyceride Secretion and Reduces Hepatic Lipid Content by Promoting Very Low Density Lipoprotein Particle Expansion. Arterioscler Thromb. Vasc. Biol. 33 (11), 2501–2508. 10.1161/ATVBAHA.113.301948 24030551

[B48] Wang D. Liu Y. Liu S. Lin L. Liu C. Shi X. (2018). Serum Fetuin-B Is Positively Associated with Intrahepatic Triglyceride Content and Increases the Risk of Insulin Resistance in Obese Chinese Adults: A Cross-Sectional Study. J. Diabetes 10 (7), 581–588. 10.1111/1753-0407.12632 29194974

[B49] Wang F. Kohan A. B. Lo C.-M. Liu M. Howles P. Tso P. (2015). Apolipoprotein A-IV: A Protein Intimately Involved in Metabolism. J. Lipid Res. 56 (8), 1403–1418. 10.1194/jlr.R052753 25640749 PMC4513983

[B50] Xin H. Zhang X. Sun D. Zhang C. Hao Y. Gu X. (2018). Chronic Heat Stress Increases Insulin-Like Growth factor-1(IGF-1) but Does Not Affect IGF-Binding Proteins in Growing Pigs. J. Therm. Biol. 77, 122–130. 10.1016/j.jtherbio.2018.08.008 30196890

[B51] Zhou Q. Zhao L. Shao Z. Declerck P. Leung L. L. K. Morser J. (2022). Both plasma Basic Carboxypeptidases, Carboxypeptidase B2 and Carboxypeptidase N, Regulate Vascular Leakage Activity in Mice. J. Thromb. Haemost 20 (1), 238–244. 10.1111/jth.15551 34626062

[B52] Zhou W. Yang J. Zhu J. Wang Y. Wu Y. Xu L. (2019). Fetuin B Aggravates Liver X Receptor-Mediated Hepatic Steatosis through AMPK in HepG2 Cells and Mice. Am. J. Transl Res. 11 (3), 1498–1509. 30972177 PMC6456555

