# Supplementary material for: Acute Endoplasmic Reticulum Stress Induces Inflammation Reaction, Complement System Activation, and Lipid Metabolism Disorder of Piglet Livers: A Proteomic Approach
Source: Front Physiol. 2022 Apr 13;13:857853. doi: 10.3389/fphys.2022.857853 (PMC9043290; doi:10.3389/fphys.2022.857853)
Supplement: Supplementary file 2 [file DataSheet2.docx]

Supplementary Material

# Supplementary Figures and Tables

## Supplementary Figures

*
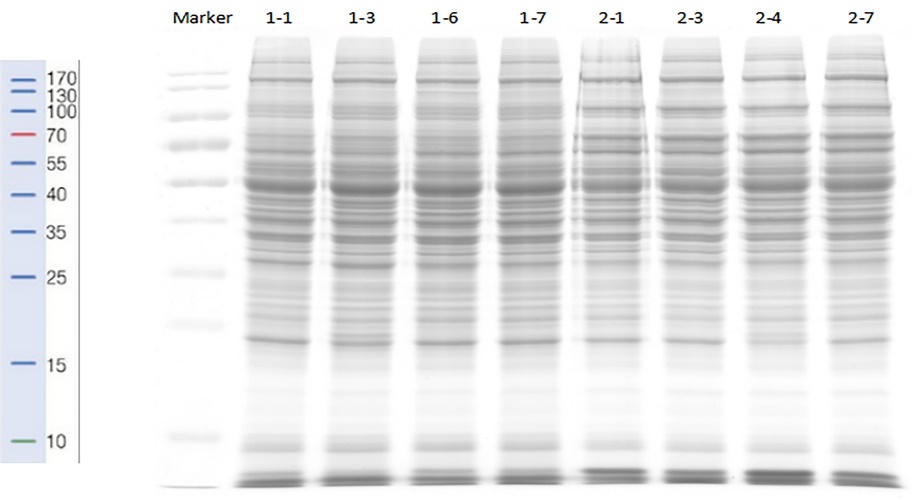
*

**Supplementary Figure 1.** SDS-PAGE gel imaging of experimental samples: electrophorese the eight samples and marker. The first four samples were vehicle group samples, and the last four samples were TM group samples.


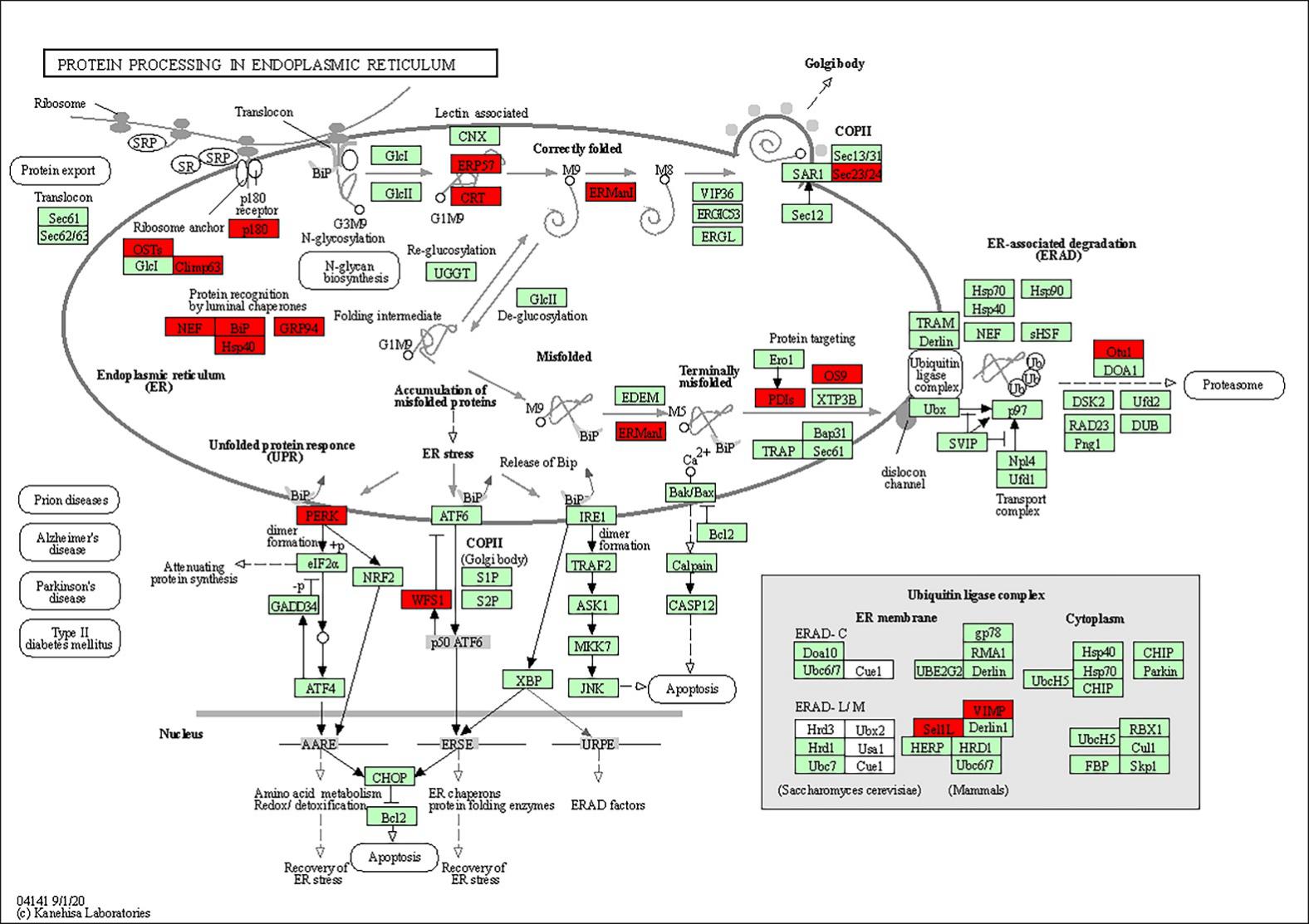


**Supplementary Figure 2.** Protein processing in the endoplasmic reticulum pathway chart: compared with the control group, red boxes are the nodes of DEPs, and green boxes are the genes or enzymes specific to the species. The numbers in the box represent the enzyme number (EC number), and the entire pathway is composed of complex biochemical reactions catalyzed by multiple enzymes. The enzyme nodes associated with the DEPs in this pathway diagram are highlighted in red.


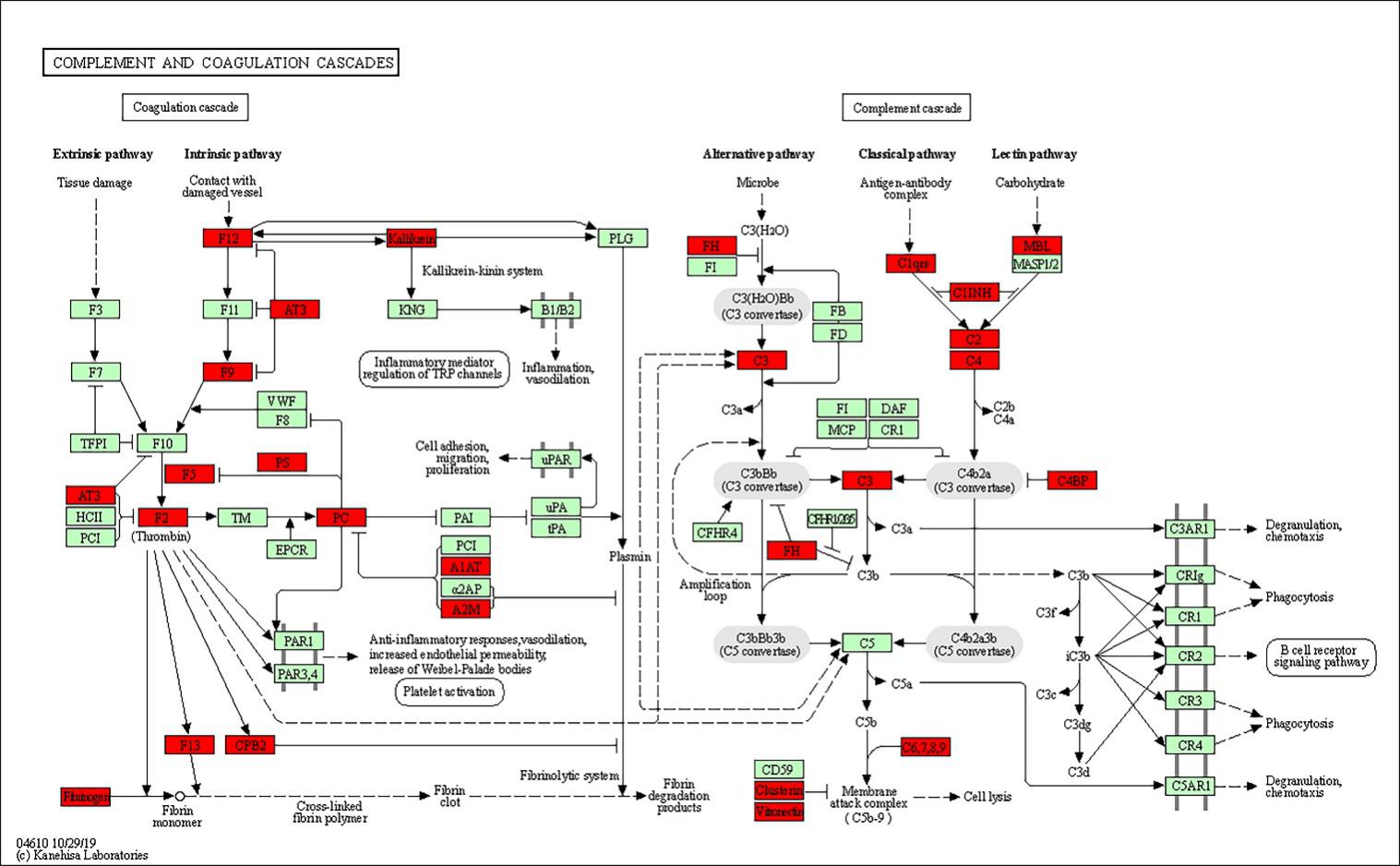


**Supplementary Figure 3.** Complement coagulation cascade pathway chart: compared with the control group, red boxes are the nodes of DEPs, and green boxes are the genes or enzymes specific to the species. The numbers in the box represent the EC number, and the entire pathway is composed of complex biochemical reactions catalyzed by multiple enzymes. The enzyme nodes associated with the DEPs in this pathway diagram are highlighted in red.

# Supplementary Tables

**Table S1. Specific primer sequences used in quantitative real-time PCR analysis**

| Gene name | Genebank | Primer (5’-3’) | Product (bp) |
| --- | --- | --- | --- |
| GRP94 | NM_214103.1 | F: ACACTGCGGTCAGGGTAT  R: TTCTTCGTCGTCGTCTTG | 180 |
| GRP78 | XM_001927795.7 | F: AATGGCCGTGTGGAGATCA  R: GAGCTGGTTCTTGGCTGCAT | 114 |
| ATF6 | XM_021089515.1 | F: CCAGTCCTTGCTGTCACT  R: CACACTTCTCATGGTACTCTG | 141 |
| ATF4 | NM_001123078.1 | F: GCCCCCGCAGATAGTGAA  R: TGGGGAAAGGGGAAGAGTTTG | 103 |

**Table S2.** Sample protein concentration

| **sample name** | **OD values** | **remove the background** | **Post-dilution concentration（μg/μL）** | | **dilution ratio** | **the original concentration（μg/μL）** |
| --- | --- | --- | --- | --- | --- | --- |
| 1_1 | 1.2 | 0.724 | | 0.68 | 20 | 13.61 |
| 1_3 | 1.175 | 0.699 | | 0.64 | 20 | 12.89 |
| 1_6 | 1.115 | 0.639 | | 0.56 | 20 | 11.24 |
| 1_7 | 1.165 | 0.689 | | 0.63 | 20 | 12.61 |
| 2_1 | 1.272 | 0.796 | | 0.79 | 10 | 7.9 |
| 2_3 | 1.32 | 0.844 | | 0.87 | 10 | 8.68 |
| 2_4 | 1.219 | 0.743 | | 0.71 | 10 | 7.09 |
| 2_7 | 1.311 | 0.835 | | 0.85 | 10 | 8.53 |

**Table S3.** Quantitative information of protein

| **Identification of protein number** | **Quantitative protein number** | **Unique polypeptide number** | **Unique spectrum number** | **Effective spectrum rate** |
| --- | --- | --- | --- | --- |
| 3679 | 3679 | 26109 | 44892 | 26% |

**Table S4.** The specific DEPs involved in protein processing in endoplasmic reticulum pathway

| **Accession Number** | **Identified Proteins** | **Student's T-test p-value 1_2** | **Ratio(2/1)** |
| --- | --- | --- | --- |
| XP_020955243.1 | protein sel-1 homolog 1 isoform X1 [Sus scrofa] | 0.000155551 | 0.739989198 |
| XP_020947548.1 | LOW QUALITY PROTEIN: protein OS-9 [Sus scrofa] | 0.00141329 | 0.742866833 |
| XP_003132136.3 | dolichyl-diphosphooligosaccharide--protein glycosyltransferase subunit STT3B [Sus scrofa] | 0.000776908 | 0.743001998 |
| NP_999484.1 | interferon-induced, double-stranded RNA-activated protein kinase [Sus scrofa] | 0.00655814 | 0.783882721 |
| XP_003482499.3 | protein transport protein Sec24D [Sus scrofa] | 0.0072164 | 1.20494602 |
| XP_013835092.1 | ubiquitin thioesterase OTU1 isoform X1 [Sus scrofa] | 0.0444567 | 1.210279308 |
| XP_005668217.2 | dnaJ homolog subfamily C member 1 [Sus scrofa] | 0.034231 | 1.221503384 |
| XP_020933695.1 | ribosome-binding protein 1 isoform X1 [Sus scrofa] | 0.00177866 | 1.22314404 |
| NP_001167604.1 | calreticulin precursor [Sus scrofa] | 0.00570881 | 1.225794707 |
| NP_001182048.1 | protein disulfide-isomerase A6 precursor [Sus scrofa] | 0.00136635 | 1.301783685 |
| XP_020922086.1 | protein disulfide-isomerase [Sus scrofa] | 0.00341097 | 1.333110428 |
| XP_020956467.1 | LOW QUALITY PROTEIN: wolframin [Sus scrofa] | 0.0102195 | 1.344959796 |
| NP_001231560.1 | cytoskeleton-associated protein 4 [Sus scrofa] | 0.0155905 | 1.374052769 |
| NP_001157585.1 | selenoprotein S [Sus scrofa] | 0.00961293 | 1.37827064 |
| XP_020931971.1 | dnaJ homolog subfamily C member 10 [Sus scrofa] | 0.000329553 | 1.4031466 |
| XP_020936709.1 | ERmannosyl-oligosaccharide 1,2-alpha-mannosidase [Sus scrofa] | 0.000182418 | 1.418465177 |
| NP_001182041.1 | protein disulfide-isomerase A3 precursor [Sus scrofa] | 3.87085E-05 | 1.427876895 |
| NP_001231275.1 | dnaJ homolog subfamily B member 11 precursor [Sus scrofa] | 0.000438897 | 1.447340172 |
| NP_999268.1 | endoplasmin precursor [Sus scrofa] | 1.68854E-05 | 1.835915543 |
| NP_001254763.1 | protein disulfide-isomerase A4 precursor [Sus scrofa] | 5.39836E-05 | 1.881307074 |
| NP_001177113.1 | dnaJ homolog subfamily C member 3 precursor [Sus scrofa] | 5.55996E-06 | 1.922198604 |
| XP_005667476.2 | hypoxia up-regulated protein 1 isoform X3 [Sus scrofa] | 1.20019E-05 | 2.141280738 |
| XP_001927830.4 | 78 kDa glucose-regulated protein isoform X2 [Sus scrofa] | 2.12629E-05 | 2.278994081 |

**Table S5.** The specific DEPs involved in metabolic pathway

| **Accession Number** | **Identified Proteins** | **Student's T-test p-value 1_2** | **Ratio(2/1)** |
| --- | --- | --- | --- |
| XP_003357857.2 | putative 2-oxo-4-hydroxy-4-carboxy-5-ureidoimidazoline decarboxylase [Sus scrofa] | 0.0344945 | 0.558314822 |
| NP_001123420.1 | L-gulonolactone oxidase [Sus scrofa] | 0.00140024 | 0.593357432 |
| NP_999586.1 | cytochrome P450 2E1 [Sus scrofa] | 0.0196221 | 0.610991898 |
| XP_013853463.2 | LOW QUALITY PROTEIN: UDP-glucuronosyltransferase 2B31 [Sus scrofa] | 0.00155647 | 0.61139 |
| NP_001093400.1 | fatty acid synthase [Sus scrofa] | 0.00253429 | 0.632930945 |
| NP_999597.1 | lanosterol 14-alpha demethylase [Sus scrofa] | 2.49065E-05 | 0.65178886 |
| XP_003357000.2 | LOW QUALITY PROTEIN: UDP-glucuronosyltransferase 2B31 [Sus scrofa] | 0.014335 | 0.655909148 |
| XP_020957564.1 | serum paraoxonase/arylesterase 1 isoform X1 [Sus scrofa] | 0.0375317 | 0.680155435 |
| XP_020941206.1 | cytochrome P450, subfamily IIIA, polypeptide 22 isoform X1 [Sus scrofa] | 0.0128461 | 0.694511984 |
| XP_003129115.1 | UDP-glucuronosyltransferase 2C1 [Sus scrofa] | 0.0160501 | 0.696714871 |
| NP_001239144.1 | hydroxymethylglutaryl-CoA synthase, cytoplasmic [Sus scrofa] | 0.00223685 | 0.702582363 |
| XP_005657566.1 | cytochrome P450 2E1 isoform X1 [Sus scrofa] | 0.00722952 | 0.705374953 |
| NP_001033783.1 | long-chain-fatty-acid--CoA ligase 4 [Sus scrofa] | 0.00661563 | 0.708303046 |
| NP_001153086.1 | cytochrome P450 1A2 [Sus scrofa] | 0.02012 | 0.730056746 |
| NP_001295402.1 | aldehyde oxidase [Sus scrofa] | 0.00479438 | 0.730898141 |
| XP_003132136.3 | dolichyl-diphosphooligosaccharide--protein glycosyltransferase subunit STT3B [Sus scrofa] | 0.000776908 | 0.743001998 |
| XP_003122689.1 | triokinase/FMN cyclase [Sus scrofa] | 0.000530075 | 0.749751524 |
| NP_001001641.1 | bifunctional epoxide hydrolase 2 [Sus scrofa] | 0.000522499 | 0.753987759 |
| NP_001161307.1 | cytochrome P450 2C42 precursor [Sus scrofa] | 0.0265862 | 0.755429505 |
| XP_020945381.1 | pyruvate kinase PKLR isoform X2 [Sus scrofa] | 0.0118702 | 0.759491194 |
| NP_999209.1 | dihydropyrimidine dehydrogenase [NADP(+)] precursor [Sus scrofa] | 0.0015516 | 0.760105486 |
| NP_001137167.1 | acetyl-coenzyme A synthetase, cytoplasmic [Sus scrofa] | 0.0157112 | 0.761383617 |
| XP_005656211.1 | adenylate kinase 4, mitochondrial isoform X1 [Sus scrofa] | 0.0307609 | 0.762379503 |
| XP_003128019.3 | phospholipid phosphatase 3 isoform X1 [Sus scrofa] | 0.014118 | 0.769799521 |
| XP_013835161.1 | serum paraoxonase/arylesterase 2 isoform X1 [Sus scrofa] | 0.00177368 | 0.770776004 |
| XP_005662058.1 | phosphoserine phosphatase isoform X1 [Sus scrofa] | 0.00083891 | 0.772876799 |
| XP_020920809.1 | LOW QUALITY PROTEIN: phosphoserine aminotransferase [Sus scrofa] | 0.0107798 | 0.778192844 |
| NP_001191700.1 | S-methylmethionine--homocysteine S-methyltransferase BHMT2 [Sus scrofa] | 0.0427186 | 0.779570685 |
| XP_005672351.1 | UDP glucuronosyltransferase 1 family, polypeptide A6 isoform X2 [Sus scrofa] | 0.0168602 | 0.779816208 |
| XP_020950195.1 | very-long-chain 3-oxoacyl-CoA reductase-B-like [Sus scrofa] | 2.22335E-05 | 0.784381716 |
| NP_001230283.1 | delta(24)-sterol reductase [Sus scrofa] | 0.0398544 | 0.784629885 |
| XP_020949325.1 | diphosphomevalonate decarboxylase [Sus scrofa] | 0.000759218 | 0.788991402 |
| XP_020918083.1 | LOW QUALITY PROTEIN: ribonucleoside-diphosphate reductase large subunit [Sus scrofa] | 7.24383E-06 | 0.789832919 |
| NP_001265679.1 | UDP glucuronosyltransferase 1 family, polypeptide A6 precursor [Sus scrofa] | 0.0186238 | 0.799004625 |
| XP_020938823.1 | very-long-chain 3-oxoacyl-CoA reductase isoform X1 [Sus scrofa] | 0.0117346 | 0.800108649 |
| XP_003482212.1 | glutamate--cysteine ligase catalytic subunit isoform X1 [Sus scrofa] | 0.0277923 | 0.806207664 |
| XP_005668301.1 | isopentenyl-diphosphate Delta-isomerase 1 isoform X2 [Sus scrofa] | 0.0018347 | 0.807039095 |
| XP_005656566.2 | UDP-glucuronosyltransferase 2A3 isoform X1 [Sus scrofa] | 0.028499 | 0.80856999 |
| XP_020919537.1 | glycine dehydrogenase (decarboxylating), mitochondrial [Sus scrofa] | 0.00255414 | 0.80862641 |
| XP_020957345.1 | ethanolamine-phosphate phospho-lyase isoform X1 [Sus scrofa] | 0.0232673 | 0.813071053 |
| NP_001231396.1 | succinate-semialdehyde dehydrogenase, mitochondrial [Sus scrofa] | 0.0252161 | 0.816471177 |
| NP_999074.1 | glutamine synthetase [Sus scrofa] | 0.00481556 | 0.816561068 |
| XP_020940139.1 | dimethylglycine dehydrogenase, mitochondrial isoform X1 [Sus scrofa] | 0.0442441 | 0.819278838 |
| XP_020948901.1 | beta,beta-carotene 15,15'-dioxygenase isoform X1 [Sus scrofa] | 0.00760958 | 0.821405043 |
| NP_001182259.1 | catechol O-methyltransferase [Sus scrofa] | 0.00571686 | 0.821560212 |
| XP_005671322.2 | pantothenate kinase 1 isoform X1 [Sus scrofa] | 0.047594 | 0.821907841 |
| XP_003355507.1 | 17-beta-hydroxysteroid dehydrogenase type 6 [Sus scrofa] | 0.0490406 | 0.827010017 |
| XP_005662547.2 | glutamine--fructose-6-phosphate aminotransferase [isomerizing] 1 isoform X1 [Sus scrofa] | 0.00927998 | 1.202532826 |
| NP_001172068.1 | sialic acid synthase [Sus scrofa] | 1.08828E-05 | 1.203876972 |
| XP_003130728.1 | beta-1,4-galactosyltransferase 1 [Sus scrofa] | 0.0178104 | 1.208537911 |
| NP_001123442.1 | cytochrome c [Sus scrofa] | 0.00148654 | 1.215311223 |
| NP_001335893.1 | 4-hydroxyphenylpyruvate dioxygenase [Sus scrofa] | 0.0192998 | 1.228432402 |
| XP_005672216.2 | LOW QUALITY PROTEIN: carbamoyl-phosphate synthase [ammonia], mitochondrial [Sus scrofa] | 0.000125877 | 1.27966135 |
| XP_013849657.1 | DNA-directed RNA polymerase II subunit RPB7 [Sus scrofa] | 0.000996631 | 1.285905654 |
| NP_001116691.1 | 2-oxoisovalerate dehydrogenase subunit beta, mitochondrial [Sus scrofa] | 0.00099194 | 1.294000972 |
| NP_001135459.1 | phosphatidate phosphatase LPIN2 [Sus scrofa] | 0.0002613 | 1.296234884 |
| XP_005673261.1 | alpha-aminoadipic semialdehyde synthase, mitochondrial isoform X1 [Sus scrofa] | 0.000371436 | 1.304886658 |
| XP_001927564.2 | delta-1-pyrroline-5-carboxylate synthase isoform X1 [Sus scrofa] | 0.0172096 | 1.338267313 |
| NP_001231470.1 | mannose-1-phosphate guanyltransferase beta [Sus scrofa] | 4.07049E-05 | 1.346612401 |
| NP_001172070.1 | ornithine aminotransferase, mitochondrial [Sus scrofa] | 0.00120885 | 1.401106669 |
| XP_020936709.1 | ERmannosyl-oligosaccharide 1,2-alpha-mannosidase [Sus scrofa] | 0.000182418 | 1.418465177 |
| NP_001302663.1 | cytochrome P450 2C23-like [Sus scrofa] | 0.00976253 | 1.465850337 |
| NP_999213.1 | arginase-1 [Sus scrofa] | 2.76397E-05 | 1.466795211 |
| XP_020928761.1 | bifunctional 3'-phosphoadenosine 5'-phosphosulfate synthase 2 isoform X1 [Sus scrofa] | 0.000431596 | 1.47701354 |
| XP_020927212.1 | LOW QUALITY PROTEIN: beta-galactosidase [Sus scrofa] | 0.00432863 | 1.558235187 |
| NP_001161115.1 | cysteine dioxygenase type 1 [Sus scrofa] | 6.99814E-05 | 1.660061315 |
| XP_020947867.1 | branched-chain-amino-acid aminotransferase, cytosolic isoform X1 [Sus scrofa] | 4.22482E-05 | 1.694242481 |
| XP_020929679.1 | cytochrome P450 2C42 [Sus scrofa] | 0.00175138 | 1.703298019 |
| NP_001116618.1 | nicotinamide N-methyltransferase [Sus scrofa] | 0.00472348 | 1.860600248 |
| XP_003127043.1 | alanine aminotransferase 2 [Sus scrofa] | 0.000170659 | 1.92998062 |
| XP_003126932.3 | tyrosine aminotransferase [Sus scrofa] | 0.000562271 | 2.254602233 |
| XP_001928714.1 | arginase-2, mitochondrial isoform X1 [Sus scrofa] | 0.00001617 | 2.727117024 |

**Table S6.** The specific DEPs involved in complement and coagulation cascade pathway

| **Accession Number** | **Identified Proteins** | **Student's T-test p-value 1_2** | **Ratio(2/1)** |
| --- | --- | --- | --- |
| NP_999269.1 | vitronectin precursor [Sus scrofa] | 0.00450426 | 0.685649738 |
| XP_001929181.1 | carboxypeptidase B2 [Sus scrofa] | 0.000165634 | 0.726644329 |
| NP_001116666.1 | plasma protease C1 inhibitor precursor [Sus scrofa] | 4.71004E-05 | 0.755362347 |
| NP_001007195.1 | mannose-binding protein A precursor [Sus scrofa] | 0.00271176 | 0.757262467 |
| NP_999136.1 | clusterin precursor [Sus scrofa] | 0.00718174 | 0.772193769 |
| NP_001116561.1 | complement C4-A precursor [Sus scrofa] | 0.00727746 | 0.802116448 |
| NP_001095285.1 | complement C2 precursor [Sus scrofa] | 0.0225703 | 0.818888621 |
| NP_001231453.1 | fibrinogen gamma chain precursor [Sus scrofa] | 0.0143975 | 1.22650461 |
| XP_005654909.1 | complement C3 [Sus scrofa] | 0.00862328 | 1.23859669 |
| NP_001123430.1 | antithrombin-III precursor [Sus scrofa] | 0.000298803 | 1.243703692 |
| NP_001157475.1 | coagulation factor IX [Sus scrofa] | 0.00062294 | 1.248020236 |
| NP_001335871.1 | alpha-1-antitrypsin precursor [Sus scrofa] | 0.0210183 | 1.271451882 |
| NP_001005349.1 | complement C1s subcomponent precursor [Sus scrofa] | 3.2038E-06 | 1.278516516 |
| XP_020957142.1 | fibrinogen alpha chain isoform X1 [Sus scrofa] | 0.00354604 | 1.29629739 |
| XP_020948067.1 | complement C1r subcomponent [Sus scrofa] | 0.0253299 | 1.317553883 |
| XP_020926395.1 | vitamin K-dependent protein S [Sus scrofa] | 0.000347304 | 1.325527728 |
| NP_999083.1 | vitamin K-dependent protein C precursor [Sus scrofa] | 0.000269155 | 1.337751074 |
| NP_999446.1 | complement factor H precursor [Sus scrofa] | 0.000228673 | 1.348852595 |
| XP_001927665.3 | coagulation factor XIII A chain [Sus scrofa] | 0.00583065 | 1.380695086 |
| NP_999239.1 | plasma kallikrein [Sus scrofa] | 0.000233237 | 1.413394795 |
| XP_013843850.2 | alpha-2-macroglobulin isoform X1 [Sus scrofa] | 0.000526312 | 1.423214932 |
| XP_005667607.2 | C4b-binding protein alpha chain isoform X1 [Sus scrofa] | 0.00886613 | 1.439829252 |
| NP_999447.1 | complement component C7 precursor [Sus scrofa] | 0.00345853 | 1.474277776 |
| NP_999285.1 | coagulation factor V precursor [Sus scrofa] | 1.14325E-05 | 1.503192859 |
| NP_999407.1 | coagulation factor XII precursor [Sus scrofa] | 9.95028E-05 | 1.507433031 |
| NP_001116457.1 | prothrombin precursor [Sus scrofa] | 0.000193082 | 1.588099351 |
| NP_001231042.1 | fibrinogen beta chain precursor [Sus scrofa] | 0.000199938 | 1.682350372 |
| XP_020948034.1 | LOW QUALITY PROTEIN: pregnancy zone protein [Sus scrofa] | 0.000418238 | 2.049845824 |
